# Multifunctional hydrogel promotes rotator cuff healing through anti-inflammation and vascularization

**DOI:** 10.1016/j.mtbio.2025.102016

**Published:** 2025-06-23

**Authors:** Bitao Wang, Yiyang Hou, Xi Shang, Yuxuan Zhou, Yubiao Yang, Zhenhan Li, Boyuan Ma, Zhi Zeng, Jinyu Chen, Cheng Tang, Jian Hao, Lianyong Wang, Xianhu Zhou

**Affiliations:** a Ningbo University Health Science Center, No. 818, Fenghua Road, Jiangbei District, Ningbo, Zhejiang, 315211, China; b Key Laboratory of Bioactive Materials, Ministry of Education, College of Life Sciences, Nankai University, Tianjin, 300071, China; c Taizhou Hospital of Zhejiang Province affiliated to Wenzhou Medical University, No.1 Tong Yang Road East, Luqiao District, Taizhou City, Zhejiang Province, 318050, China; d Portola High School, 1001 Cadence, Irvine, CA, 92618, United States; e The Second Affiliated Hospital of Guangzhou Medical University, Guangzhou, 510260, China

**Keywords:** Schiff base, Macrophage polarization, Rotator cuff tear, Anti-inflammation, Vascularization

## Abstract

Rotator cuff tears (RCTs) represent a substantial clinical challenge due to their intricate, multi-stage healing process, which encompasses sequential phases of inflammation, proliferation, tissue reconstruction, and remodeling. Disruption of any phase can adversely affect healing and increase the risk of re-tear. To address this issue, we engineered an injectable LA-CMCS-OHA hydrogel by physically blending lactic acid (LA)—a key signaling molecule—into a composite hydrogel system consisting of carboxymethyl chitosan (CMCS) and oxidized hyaluronic acid (OHA). The hydrogel formation was facilitated by a dynamic Schiff base reaction between CMCS and OHA, imparting superior biocompatibility, injectability, and self-healing properties. The LA-CMCS-OHA hydrogel effectively inhibits nuclear factor kappa B (NF-κB) signaling, thereby promoting macrophage polarization toward the anti-inflammatory M2 phenotype and mitigating early inflammatory responses. Additionally, it markedly enhances vascular endothelial growth factor (VEGF) expression, stimulating the proliferation and migration of human umbilical vein endothelial cells (HUVECs) to facilitate neovascularization. These therapeutic effects were comprehensively validated through both in *vitro* and in *vivo* studies. Histological and biomechanical assessments using a rat acute RCT model revealed that the LA-CMCS-OHA hydrogel significantly facilitates tendon-bone regeneration. Collectively, these findings highlight the LA-CMCS-OHA hydrogel as a promising therapeutic strategy for RCTs with strong potential for clinical translation.

## Highlights

• Lactic acid is a significant signalling molecule involved in tissue repair and disease development.
• LA-CMCS-OHA hydrogel promotes macrophage polarization toward M2 anti-inflammatory phenotype by inhibiting NF-κB pathway.
• The LA-CMCS-OHA hydrogel enhances vascular endothelial growth factor secretion, thereby facilitating neovascularization.
• The LA-CMCS-OHA hydrogel creates a microenvironment for RCT repair, showing huge potential for clinical translation.

## Graphical abstract

Schematic diagram illustrates the regenerative process of LA-CMCS-OHA hydrogel for treating rotator cuff tears. (A) Preparation of the LA-CMCS-OHA hydrogel. (B) The LA-CMCS-OHA hydrogel mitigates the inflammatory response via the NF-κB pathway, stimulates VEGF secretion, promotes endothelial cell proliferation and vascularization, and establishes a favorable microenvironment for tendon-bone reconstruction.

**Figure:**
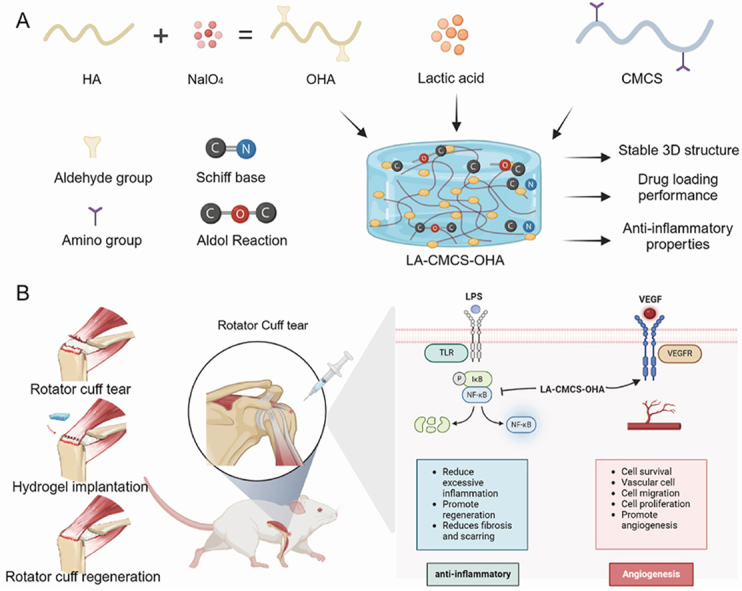

## 1 Introduction

RCT is one of the most common musculoskeletal disorders and its occurrence is closely related to aging and shoulder involvement [1]. RCTs can be managed non-surgically using physiotherapy, nonsteroidal anti-inflammatory drugs, or injections [2]. However, surgical repair is typically recommended in most cases [3]. Despite advancements in surgical techniques, the post-surgical re-tear rate remains alarmingly high, reaching nearly 50 % [4]. Furthermore, the damaged rotator cuff rarely regains its original structure or function.

RCT healing is a complex, multi-stage process involving inflammation, proliferation, remodeling, and maturation, with any disruption potentially hindering recovery [5]. The inflammatory phase is particularly critical, initiating an immune response that recruits diverse immune cells, including neutrophils, monocytes, mast cells, and macrophages [6]. Among these, macrophages play a pivotal role by clearing cellular debris, presenting antigens, promoting angiogenesis, and secreting cytokines to regulate the healing process [7]. Macrophages are highly heterogeneous, with their polarization into distinct phenotypes determined by the local microenvironment [8]. The two primary phenotypes are the pro-inflammatory M1 and the anti-inflammatory M2 [9]. M1 macrophages produce inflammatory cytokines such as TNF-α, IL-1β, and IL-6, which contribute to tissue damage. Conversely, M2 macrophages exhibit significant anti-inflammatory properties, releasing cytokines like IL-10, IL-4, and TGF-β to support tissue repair [10]. Consequently, modulating macrophage polarization to reduce inflammation is essential for promoting rotator cuff repair.

LA plays a crucial role in cellular signaling by facilitating communication among cells, organs, and tissues. Contrary to common misconceptions, it is not merely a metabolic byproduct of hypoxic conditions [11]. Recent studies indicate that LA regulates various biological functions, including energy metabolism, immune responses, memory formation, wound healing, and cell cycle progression [12,13]. During wound healing, LA serves as an energy substrate to meet increased metabolic demands and reduces pH, which is crucial for cell proliferation and differentiation [14]. LA has been demonstrated to stimulate the secretion of VEGF, which in turn promotes angiogenesis [15]. Research further reveals that high concentrations of LA promote M2 macrophage polarization and suppression of the inflammatory response, thus enhancing tissue regeneration[[16], [17], [18]]. However, prolonged exposure to elevated LA levels may trigger a shift from acute to chronic inflammation [19], exacerbate ischemic tissue damage [20], and promote tumor growth and metastasis [21,22]. Typically, LA degrades rapidly in *vivo*, limiting its effectiveness as a signaling molecule [23]. Consequently, developing a tissue-engineered scaffold that enables sustained LA release for therapeutic benefits is essential.

Hydrogels are three-dimensional, cross-linked networks of water-soluble polymers, effectively encapsulating drugs, bioactive factors, proteins, and cells. Hydrogels are frequently utilized in controlled drug delivery applications due to their excellent biocompatibility and biodegradability [24]. For example, inflammation-responsive core-shell micro-hydrogels, through their sustained release of SDF-1 and IL-4, have been shown to effectively enhance tendon-bone healing [25]. Similarly, macroporous granular hydrogels containing small extracellular vesicles have demonstrated efficacy in promoting osteoporotic tendon-bone healing [26]. Hyaluronic acid(HA), a naturally abundant polysaccharide in human tissues, plays a pivotal role in vital physiological processes, such as joint lubrication, regulation of blood vessel permeability, and wound healing, due to its integral presence in the extracellular matrix [27]. OHA was synthesized by oxidising HA, encompasses reactive aldehyde groups, allowing for the formation of stable, three-dimensional network hydrogels [28]. Research indicates that OHA enhances wound healing by promoting M2 macrophage polarization and reducing inflammation [29,30]. Derived from chitin through deacetylation, chitosan is celebrated for its exceptional biocompatibility and biodegradability in biomedicine, although its limited solubility in neutral aqueous solutions may curtail broader applications [31]. CMCS, a derivative of chitosan, overcomes this issue and is preferred in hydrogel formulations due to its superior biocompatibility, biodegradability, antibacterial properties, and low cytotoxicity [32]. Studies demonstrate that CMCS-based hydrogels attenuate inflammatory responses by fostering macrophage polarization [33,34]. The composite hydrogel, consisting of CMCS and OHA, exhibits a similar capacity to mitigate inflammation through macrophage polarization [35,36].

In this study, LA was incorporated into a composite hydrogel composed of CMCS and OHA via physical mixing. The resulting LA-CMCS-OHA hydrogel exhibited notable self-healing properties and injectability, enabling direct application to the RCT site. The findings indicate that the hydrogel inhibits the NF-κB pathway, promotes macrophage polarization toward the M2 anti-inflammatory phenotype, and attenuates early inflammatory responses. Meanwhile, the hydrogel enhances VEGF secretion, thereby facilitating neovascularization. In a rat model of acute RCT, the LA-CMCS-OHA hydrogel created a favorable microenvironment for rotator cuff repair by reducing inflammation and restoring blood supply, ultimately promoting tissue regeneration. This innovative tissue engineering scaffold emphasizes local immunomodulation and vascularization, offering promising prospects for clinical translation.

## 2 Materials and methods

### 2.1 Materials and cells

HA (Mn = 40-100 KDa) was purchased from Macklin (Shanghai, China). CMCS (carboxymethylation ≥80 %) was obtained from Merck (Darmstadt, Germany). Sodium periodate (NaIO4) and ethylene glycol were supplied by Aladdin (Shanghai, China). LA and lipopolysaccharide (LPS) were supplied by Sigma-Aldrich. Fetal bovine serum (FBS), Dulbecco's Modified Eagle Medium (DMEM), and penicillin–streptomycin (PS) solution were obtained from Gibco BRL. The Cell Counting Kit-8 (CCK-8), Live/Dead Cell Staining Kit (AM-PI) and EdU Cell Proliferation Kit were purchased from Beyotime (Shanghai, China). ELISA kits were acquired from Beijing Solarbio Technology Co., Ltd (Beijing, China). Matrigel was obtained from Guangzhou Shuoheng Biotechnology Co (Guangzhou, China). FastPure® Cell Total RNA Isolation Kit V2, PrimeScriptTM RT reagent Kit and SYBR® Premix Ex TaTM were Purchased from Vazyme (Nanjing, China). RAW 264.7 cells and HUVEC were sourced from Cybkon Biotechnology Co., LTD (Shanghai, China).

### 2.2 Biocompatibility of LA

To evaluate the cytotoxicity of LA, RAW 264.7 cells and HUVECs were cultured in 96-well plates at a density of 8 × 10^3^ cells per well and exposed to varying LA concentrations, ranging from 1 mM to 30 mM. During the specified experimental period, the cells were incubated in a complete culture medium supplemented with 10 % CCK-8 for 2 h. Subsequently, the absorbance of each treatment group was measured at 450 nm using an enzymatic microplate reader (BioTek Inc., USA). The experiment was replicated six times, with each repetition utilizing independently prepared samples.

### 2.3 Synthesis of OHA

OHA was prepared according to the method of Zhou et al. with slight modifications [37]. Briefly, 2.5 g of HA was dissolved in 100 mL of distilled water until completely dissolved, and then 2.5 g of NaIO_4_ was added while stirring. The reaction was carried out in a dark environment at room temperature for 12 h. The oxidation reaction was terminated by adding 3.5 mL of ethylene glycol drop by drop, and stirring was continued for 30 min. The resulting product was dialyzed in an 8000–14000 Da dialysis bag for 3 days to remove the by-products. Finally, the dialysate was freeze-dried to obtain OHA. The oxidized structure and degree of oxidation of OHA were demonstrated by ^1^H NMR hydrogen spectroscopy, and the oxidation of OHA was retested by hydroxylamine hydrochloride titration to determine the aldehyde content [38]. The oxidation degree of OHA was about 53 %.

### 2.4 Synthesis of LA-CMCS-OHA hydrogel

The 1.5 % CMCS, 3 % CMCS, 4.5 % CMCS, and 45 mM LA solutions were prepared using deionized water as solvent. The 9 % OHA solution was prepared and heated in a water bath at 60 °C until a clear and homogeneous solution was obtained, and left to cool to room temperature. The different concentrations of CMCS solution, OHA solution, and LA solution were mixed in equal volumes, stirred at 1500 rpm for 5 min, and left to stand to obtain the hydrogel. The composite hydrogel systems were named LA-1.5CMCS-OHA, LA-3CMCS-OHA, and LA-4.5CMCS-OHA according to the different concentrations of CMCS solution.

### 2.5 Characterization of LA-CMCS-OHA hydrogel

The three groups of LA-1.5CMCS-OHA, LA-3CMCS-OHA, and LA-4.5CMCS-OHA hydrogels were lyophilized by a freeze dryer (SCIENTZ-10ND, China). The lyophilized samples were glued to the sample stage with conductive adhesive. To improve the electrical conductivity of the samples, a thin platinum layer was sprayed on the cross-sectional surfaces of the samples, and the microscopic morphology of the samples was observed using the scanning electron microscope (FEI Apreo S LoVac, Czech Republic). The FT-IR spectra of HA, OHA, CMCS, LA-1.5CMCS-OHA, LA-3CMCS-OHA, and LA-4.5CMCS-OHA were obtained by scanning in the range of 4000-400 cm-1 with a Fourier Transform Infrared Spectrometer (Bruker TENSOR II, Germany).

### 2.6 Swelling behavior of LA-CMCS-OHA hydrogel

Since LA-1.5CMCS-OHA is in liquid form, only LA-3CMCS-OHA and LA-4.5CMCS-OHA were selected for subsequent characterization. LA-3CMCS-OHA and LA-4.5CMCS-OHA were prepared, and the lyophilized hydrogels were weighed. The pre-weighed hydrogels were soaked in phosphate-buffered saline (PBS) at 37 °C, pH = 7.4, removed every 2 h, absorbed the excess surface water with filter paper, weighed, and recorded until the swelling was equilibrated. The swelling ratio of hydrogel was calculated according to Eq. (1):

(1) Swellingratio(%)=(Ww−Wd)/Wd×100%

where Ww and Wd represent the initial weight of the lyophilized hydrogels and the weight after swelling equilibrium, respectively. The tests were carried out in triplicate.

### 2.7 Degradation of LA-CMCS-OHA hydrogel

For the in *vitro* degradation study, hydrogel samples were immersed in 2 mL of PBS at pH 6.4 or 7.4 in 5 mL test tubes and incubated at 37 °C. The initial weight (W0) of each hydrogel was recorded. At predetermined time points, hydrogels were removed, blotted dry with filter paper to remove excess surface fluid, and their remaining weight (Wt) was measured. The *vitro* degradation ratio of the hydrogel was calculated according to Eq. (2):

(2) Degradationratio(%)=(W0–Wt)/W0×100%

In *vivo* degradation experiments were conducted using female Wistar rats (Ningbo University Laboratory Animal Center). All animal procedures adhered to the guidelines of the Guide for the Care and Use of Laboratory Animals, National Research Council, United States. Surgical procedures involved shaving the rats' dorsal region and creating a 1.5 cm longitudinal skin incision for subcutaneous implantation of the LA-CMCS-OHA hydrogel. The initial weight (Wi) of the hydrogel was recorded. At 3, 5, and 7 days post-implantation, hydrogels were retrieved by reopening the original incision. After retrieval, hydrogels were blotted dry with filter paper to remove excess surface fluid, and their remaining weight (Wr) was measured. The *vivo* degradation ratio of the hydrogel was calculated according to Eq. (3):

(3) Degradationratio(%)=(Wi–Wr)/Wi×100%

### 2.8 Rheological testing

The rheological properties of the hydrogel were tested with a TA rheometer (TA Discovery 170 HR-2, USA), and the changes of the storage modulus (G′) and loss modulus (G″) of the hydrogel were investigated. The hydrogels were placed on the lower plate of the rheometer at 37 °C, using a 20 mm parallel plate fixture with a 1 mm gap between the parallel plate measurement cells. The shear viscosity was measured at shear rates from 0.1 to 100 s^−1^. The linear viscoelastic test was performed at a fixed angular frequency of 10 rad/s. The dynamic frequency sweep was carried out at a fixed strain of 1 %, and the frequency was between 0.01 and 10 rad/s. The strain was increased from 1 % to 1000 % and then reduced from 1000 % to 1 % for the step-strain rheological experiment. During the experiments, a ring of low-viscosity silicone oil was dripped around the gel samples and placed on the lower plate of the rheometer to prevent water evaporation.

### 2.9 Cell viability of LA-CMCS-OHA hydrogel

In *vitro* biocompatibility tests were performed by cultivating cells in culture media containing material extracts. The culture media comprised 90 % v/v DMEM, 10 % v/v FBS, and 0.1 % v/v penicillin-streptomycin. Material extracts were prepared by incubating 100 mg of LA-CMCS-OHA hydrogel in 1 mL of culture media at 37 °C for 24 h. The control media, composed of media without hydrogel, was used as the blank control. RAW 264.7 cells were seeded at a density of 8 × 10^3^ cells per well in a 96-well plate, with 100 μL of culture media added to each well. Following a 24 h incubation in a sterile CO_2_ incubator at 37 °C, the original culture media were replaced with material extracts diluted to concentrations of 0 %, 25 %, 50 %, 75 %, and 100 %.

The assessment of cell viability was conducted by means of the CCK-8 assay. For this, 10 μl of CCK-8 solution was added to each well, followed by a 2 h incubation at 37 °C. Subsequent to this, the absorbances at 450 nm were measured using an enzymatic microplate reader. Each experimental condition was tested in six replicates, with independently prepared samples to ensure reproducibility.

In furtherance of the evaluation of cell viability and toxicity, live/dead staining was conducted after 1, 3, and 5 days of incubation with the material extracts. The staining solution, composed of calcein-AM and PI, was added to the wells of a 12-well plate and incubated for 15 min. Fluorescent images of the stained cells were obtained using an inverted fluorescence microscope (Wetzlar, Germany) and analyzed using ImageJ software.

### 2.10 Assessing the effect of LA-CMCS-OHA hydrogel on macrophag polarization

To evaluate the effects of LA-CMCS-OHA hydrogel on macrophage polarization, we conducted experiments using Western blotting, quantitative real-time PCR (qRT-PCR), and immunofluorescence. RAW 264.7 cells were seeded in 6-well plates at a density of 5 × 10^5^ cells per well and incubated for 24 h. Macrophages were treated with 200 ng/mL LPS [39] and LA-CMCS-OHA hydrogel for 24 h. Untreated macrophages served as the control group.

For Western blot analysis, cells were lysed, and proteins were separated by SDS-PAGE, followed by electrotransfer onto PVDF membranes (0.45 μm, Millipore). Membranes were blocked with 5 % nonfat dry milk for 2 h to minimize non-specific interactions and incubated overnight at 4 °C with primary antibodies specific to CD206 (Abcam, ab64693, 1ug/ml) and iNOS (Abcam, ab178945, 1:1000). Subsequently, the membranes were washed thrice for 10 min each with TBST, and then incubated with secondary antibodies for 1 h at room temperature. The protein bands were visualized using Immobilon Western HRP mix reagent (Millipore).

Total RNA was extracted using the FastPure® Cell Total RNA Isolation Kit V2 according to the manufacturer's instructions, and RNA concentrations were measured with a NanoDrop spectrophotometer. Complementary DNA (cDNA) was synthesized via reverse transcription using the PrimeScript™ RT Reagent Kit. Gene expression analysis was conducted on a QuantGene 9600 Real-Time PCR System (Bioer, China) using SYBR® Premix Ex Taq™. Quantification for target gene expression was performed using the 2−ΔΔCt method and β-actin was used as normalization control. Primer sequences are detailed in Supplementary Table 1.

For immunofluorescence analysis, cells were fixed with 4 % paraformaldehyde and permeabilized with 0.2 % Triton X-100. The cells were then blocked with 5 % bovine serum albumin (BSA) for 1 h, followed by incubation with primary antibodies for CD206 (Abcam, ab64693, 1:200) and iNOS (Abcam, ab178945, 1:500) at 4 °C overnight. After three washes with PBST, cells were incubated with goat anti-rabbit IgG in a darkroom for 1 h and subsequently stained with 4′,6-diamidino-2-phenylindole (DAPI) for 5 min. Images were captured using an Olympus inverted fluorescence microscope.

### 2.11 In *vitro* anti-inflammatory effect

To investigate the impact of LA-CMCS-OHA hydrogel in influencing the secretion of inflammatory factors by M1 macrophages, RAW 264.7 cells are spread evenly on a 6-well plate. When the cells grew to about 70 %, PBS, LPS(200 ng/ml) and LPS(200 ng/ml) + LA-CMCS-OHA hydrogel were added for intervention, and the expression levels of inflammatory factors (TNF-α, IL-6, IL-10) and the expression level of interleukin-1-receptor antagonists (IL-1Ra) were detected at the indicated time points by qRT-PCR. The specific experimental steps are described above.

The effect of LA-CMCS-OHA hydrogel on the secretion of inflammatory factors by M1 macrophages was further verified using an enzyme-linked immunosorbent assay (ELISA) as described. After inducing the polarization of M1 macrophages with 200 ng/mL of LPS, the intervention was carried out by applying LA, CMCS-OHA hydrogel, and LA-CMCS-OHA hydrogel. The results were compared with the LPS-treated positive control group. Concentrations of TNF-α, IL-1β, and IL-6 in cell supernatants were measured at specified time points using mouse-specific ELISA kits. Optical density (OD) values were recorded at 450 nm and statistically analyzed.

### 2.12 Transcriptome sequencing of LA-CMCS-OHA hydrogel-treated macrophag and validation by immunofluorescence and western blot

Transcriptome sequencing was employed to identify differential gene expression in macrophages treated with LA-CMCS-OHA hydrogel. RNA was extracted from both LPS-treated and LA-CMCS-OHA hydrogel-treated macrophages using Trizol reagent (Ambion) and sent to Personalnio Biotech (Shanghai, China) for transcriptome sequencing and analysis. Gene expression changes with a fold change greater than 2 and a P-value less than 0.05 were considered significant. Data analysis, including the identification of differentially expressed genes and conducting gene ontology (GO) and Kyoto Encyclopedia of Genes and Genomes (KEGG) analyses, was performed using the Personalnio cloud platform (https://www.genescloud.cn). The translocation of NF-κB pathway p65 (CST, #8242, 1:400) into the nucleus was assessed through immunofluorescence, while the expression of NF-κB pathway-related proteins—including phosphorylated IκBα (p-IκBα; CST, #2859, 1:1000), IκBα (CST, #4812, 1:1000), phosphorylated p65 (p-p65; CST, #3033, 1:1000), and p65 (CST, #8242, 1:1000)—were analyzed by Western blot [40]. The grayscale values of the protein bands and fluorescence intensity were semi-quantified with Image J software.

### 2.13 Evaluation of the angiogenic activity of LA-CMCS-OHA hydrogel in *vitro*

To evaluate the effect of LA-CMCS-OHA hydrogel on angiogenesis, HUVECs were cultured in 6-well plates and treated with PBS, CMCS-OHA, or LA-CMCS-OHA. VEGFA (Abcam, ab214424, 1:1000) protein expression was analyzed via Western blotting, and the experimental procedure followed established protocols. VEGF expression levels were further validated using ELISA, while endothelial nitric oxide synthase(eNOS) and VEGF expression levels were quantified by qRT-PCR according to standard protocols.

Cell proliferation assays were conducted on HUVECs using the BeyoClick EdU Cell Proliferation Kit, which employs 5-ethynyl-2′-deoxyuridine (EdU) as a synthetic analog to monitor DNA synthesis. Images were captured with an Olympus inverted fluorescence microscope. The percentage of EdU-positive cells was calculated according to Eq. (4):

(4) EdU−positivecells(%)=(EdU−positivecells,red)/(Hoechst33342−positivecells,blue)×100%

The expression level of CD31(Proteintech, 11265-1-AP, 1:200) in HUVECs was assessed using immunofluorescence staining. HUVECs were cultured in 6-well plates, treated with PBS, CMCS-OHA hydrogel, or LA-CMCS-OHA hydrogel, and then subjected to immunofluorescence staining according to established methods. Relative fluorescence intensity data were analyzed using ImageJ software. Additionally, CD31 expression levels were measured by qRT-PCR following standard protocols.

In the tube formation assay, the liquid matrix glue pre-cooled at 4 °C was inoculated into a pre-cooled 96-well plate and placed in a cell incubator for 1 h. HUVECs were cultured on polymerized Matrigel at a density of 2 × 10^4 cells per well and exposed to PBS, CMCS-OHA hydrogel, or LA-CMCS-OHA hydrogel. Cells were stained with Calcein AM after 3 and 5 h. Fluorescent imaging was performed using a Leica inverted fluorescence microscope, and the images were analyzed with Image J software.

### 2.14 Establishment of an acute RCT repair model in rats

To assess the in vivo efficacy of LA-CMCS-OHA hydrogel, we established a rat model of RCT. Wistar rats, with an average weight of 250 ± 20 g, were randomly divided into three groups: the suture group, the CMCS-OHA hydrogel group, and the LA-CMCS-OHA hydrogel group. The animals were anesthetized by an intraperitoneal injection of sodium pentobarbital at a dosage of 40 mg/kg. According to established protocols, an acute RCT model was constructed [41]. Briefly, the supraspinatus was completely severed at the tendon-bone interface(TBI), and two 0.5 mm holes were drilled above the greater tuberosity of the humeral head. Sutures were passed through the bone tunnel for fixation. Subsequently, either CMCS-OHA hydrogel or LA-CMCS-OHA hydrogel was administered to evaluate their therapeutic effects (Supplementary Fig. 1). The animals were maintained at a constant temperature of 23 ± 2 °C and euthanized by carbon dioxide asphyxiation on days 3, 14, 28, and 56 (n ≥ 4 animals at each time point) post-operation. The supraspinatus-humeral complex was collected, fixed in 4 % paraformaldehyde, decalcified, dehydrated, rendered transparent, and embedded in wax. Coronal sections were prepared for immunological examination, including immunofluorescence staining for iNOS, CD206, CD31, and α-SMA (Servicebio, GB111364-100, 1:500); immunohistochemistry for IL-6, TNF-α, and IL-10; and histopathological examination using H&E, Toluidine blue, and Masson's trichrome staining.

### 2.15 Biomechanical test

After 8 weeks, specimens of the supraspinatus-humeral complex were collected for tensile testing. The cross-sectional area of the TBI was determined using a digital vernier caliper. The biomechanical strength of the supraspinatus-humeral complex was assessed by means of the HSS-DX1000 Universal Testing Machine (Jinan Heng Rui Jin Testing Machine Co., Ltd., China). The specimen underwent a preload of 0.1 N, followed by a uniaxial tension test until failure, conducted at a constant speed of 10 mm/min. The maximum load at the point of failure was meticulously recorded. By analyzing the load-displacement curve, the stiffness of the material was accurately determined. Finally, stress was calculated by dividing the ultimate load at failure by the cross-sectional area of the material.

### 2.16 Statistical analysis

All data are presented as mean ± standard deviation (SD). The statistical analyses were conducted using GraphPad Prism 10.2 (GraphPad Software Inc., USA). In each experiment, a minimum of three independent samples were used for statistical evaluation. The level of statistical significance was determined using either one-way analysis of variance (ANOVA), in accordance with the experimental design. Subsequent analyses were conducted using the Tukey method. Statistical significance was defined as *P* < 0.05.

## 3 RESULTS and DISCUSSION

### 3.1 Evaluation of LA cytotoxicity

LA, an intrinsic metabolic byproduct and signaling molecule, can result in lactic acidosis when excessively accumulated in serum [42]. To elucidate the influence of LA concentration on cellular responses, RAW 264.7 cells and HUVECs were cultured in conditioned media supplemented with LA at concentrations ranging from 1 to 30 mM. CCK-8 assays, conducted over three days, revealed that LA concentrations below 15 mM did not significantly affect the proliferation of RAW 264.7 cells. For these cells, 2 mM LA notably enhanced proliferation, whereas concentrations of 20 mM or higher proved inhibitory (Supplementary Fig. 2A–C). Interestingly, LA concentrations below 15 mM promoted HUVEC proliferation(Supplementary Fig. 2D–F), underscoring the critical, cell-type specific, and concentration-dependent role of LA in modulating cell viability.

### 3.2 Preparation and characterization of LA-CMCS-OHA hydrogel

**Fig. 1:**
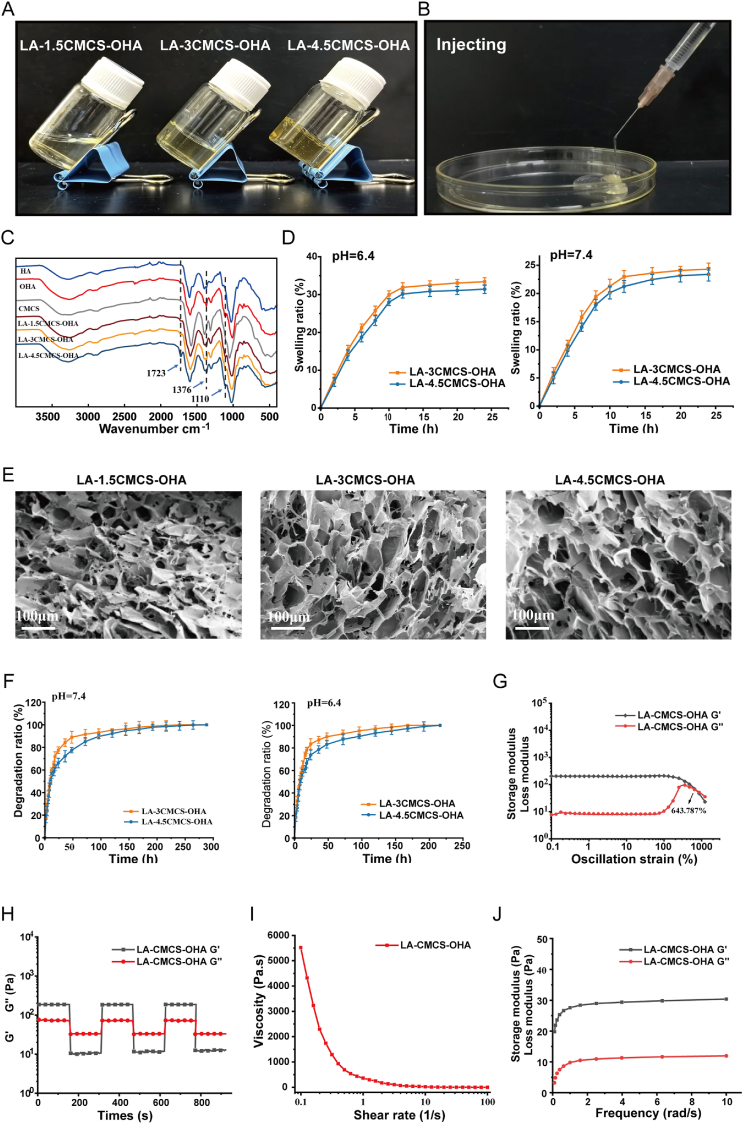
Characterization of the LA-CMCS-OHA hydrogel. (A) Digital images of LA-1.5CMCS-OHA,LA-3CMCS-OHA and LA-4.5CMCS-OHA hydrogels at room temperature. (B) Photo of LA-3CMCS-OHA hydrogel injected through a syringe. (C) FT-IR spectra of HA,OHA,CMCS,LA-1.5CMCS-OHA,LA-3CMCS-OHA and LA-4.5CMCS-OHA in the range of 4000-400 cm-1. (D) Swelling ratio of LA-3CMCS-OHA and LA-4.5CMCS-OHA hydrogels. (E) SEM images of LA-1.5CMCS-OHA,LA-3CMCS-OHA and LA-4.5CMCS-OHA hydrogels. (F) Disintegration ratio of LA-3CMCS-OHA and LA-4.5CMCS-OHA hydrogels. (G) Strain-dependent oscillatory shear rheological properties of the LA-4.5CMCS-OHA hydrogel. (H) Step-strain measurements of the LA-CMCS-OHA hydrogels three cycles at high strain (1000 %) and low strain (1 %), frequency 10 rad/s. (I) The shear viscosity of hydrogels with shear rate ranges from 0.1 to 100 s-1. (J) Dynamic frequency sweep of hydrogels.

In this study, we developed an injectable anti-inflammatory hydrogel LA-CMCS-OHA. As shown in Fig. 1A, LA-1.5CMCS-OHA was still fluid and did not form into the hydrogel, so for the subsequent experiments we focused on the LA-3CMCS-OHA and LA-4.5CMCS-OHA hydrogels. With the increase in CMCS concentration, the Schiff base interaction between the carbonyl group on OHA and the amino group carried by CMCS was enhanced and the yellow color of the gel deepened. The dynamic properties of the Schiff base also conferred good injectability to the hydrogel (Fig. 1B). The chemical structures of the hydrogels were further analyzed by FT-IR in Fig. 1C. The peak of OHA at 1723 cm-1 was the C=O stretching vibration after sodium periodate oxidation, which proved that HA was successfully oxidized. We also confirmed the oxidation of HA through 1H NMR (Supplementary Fig. 3). The successful incorporation of OHA was evidenced by the C-O-C stretching vibrational absorption peaks at 1110 cm-1 for LA-1.5CMCS-OHA, LA-3CMCS-OHA, and LA-4.5CMCS-OHA, and the characteristic C=N peak at 1376 cm-1 further indicated that the carbonyl group on the OHA was successfully linked to the amino group carried by CMCS via Schiff base.

The swelling properties of hydrogels are closely related to gas diffusion, nutrient supply, and metabolic waste removal [43]. The rotator cuff injury site is usually acidic due to inflammatory response, tissue ischemia, and hypoxia, therefore, we investigated the swelling properties of hydrogels in pH = 6.4 and 7.4 PBS solutions. As shown in Fig. 1D, the hydrogels formed by Schiff base in acidic environment showed greater swelling ability, and the swelling ability of LA-3CMCS-OHA was better than LA-4.5CMCS-OHA, which could be attributed to the increase of entanglement and interaction between molecular chains due to the excessive concentration of 4.5 % CMCS, resulting in the tendency of physical cross-linking between molecular chains to form heterogeneous regions, which affected the overall performance of the gel.

The microstructure of hydrogels is closely related to the mechanical properties [44]. The microscopic morphology of LA-1.5CMCS-OHA, LA-3CMCS-OHA, and LA-4.5CMCS-OHA was shown in Fig. 1E. The LA-3CMCS-OHA and LA-4.5CMCS-OHA hydrogels exhibited a highly porous and interconnected structure. However, since LA-1.5CMCS-OHA was in the flow state without being a gel, it could be seen that there were many microscopic cracks and fragments, with thin pore walls and loose network structure.

Suitable degradation properties of hydrogels are crucial in biomedical applications [45]. It is shown in Fig. 1F that the hydrogels were completely degraded at 288h in pH = 7.4 environment and at 216h in pH = 6.4. In both neutral and acidic conditions, the hydrogels retained more than 10 % of their mass after 72 h of incubation. Moreover, in *vivo* experiments demonstrated complete hydrogel degradation within one week(Supplementary Fig. 4). This in vivo degradation behavior corresponds with the degradation patterns observed *in vitro*. The rapid in vivo degradation aligns with the therapeutic needs for treating rotator cuff injuries, particularly during the acute inflammatory phase.

Considering the need for injectability of the hydrogel during therapy, combined with the above experimental results, we chose LA-3CMCS-OHA hydrogel for subsequent experiments and named it LA-CMCS-OHA hydrogel.

### 3.3 Rheological properties of LA-CMCS-OHA hydrogel

Since the rotator cuff TBI is subjected to mechanical stresses during joint motion, the hydrogel needs to have appropriate mechanical properties. Firstly, the storage modulus (G′) and loss modulus (G″) of the hydrogel were measured to evaluate the rheological properties of the hydrogel. It can be seen from Fig. 1G that the gel network of LA-CMCS-OHA collapsed only when the strain was >643.787 %. The step-strain test showed that the mechanical properties of LA-CMCS-OHA hydrogel could be well recovered from high to low strains, indicating that LA-CMCS-OHA hydrogel has good self-repairing properties, which is attributed to the good dynamic reversibility of Schiff base (Fig. 1H). In addition, as seen in Fig. 1I, the viscosity of LA-CMCS-OHA hydrogels gradually decreased with the increase of the shear rate, indicating that LA-CMCS-OHA hydrogels had shear-thinning and injectable properties, which was consistent with the results in Fig. 1B. Dynamic frequency scanning experiments (Fig. 1J) showed that the LA-CMCS-OHA hydrogel had a weak frequency dependence, demonstrating that the network structure was stable, which led to the stable mechanical properties of LA-CMCS-OHA hydrogel in a dynamic environment, and could satisfy the application requirements at rotator cuff injuries.

### 3.4 In *vitro* biocompatibility

To evaluate the biocompatibility of the prepared LA-CMCS-OHA hydrogel, we conducted in *vitro* cytocompatibility analysis by culturing RAW 264.7 cells in an extraction medium derived from the hydrogel. The CCK-8 assay results indicated that the LA-CMCS-OHA hydrogel did not exhibit any notable adverse effects on cellular activity over a time period of 3 days (Supplementary Fig. 5A–C). Similarly, Live/Dead staining revealed that the hydrogel exerted no significant cytotoxic effects on RAW 264.7 cells. The cell density in the hydrogel-containing medium was comparable to that of the control group. Furthermore, the cells displayed a uniform distribution, exhibited healthy growth, and some demonstrated spindle-like morphology (Supplementary Fig. 5D and E). These findings collectively confirmed that the LA-CMCS-OHA hydrogel possesses excellent biocompatibility.

### 3.5 The effect of LA-CMCS-OHA hydrogel on the polarization of macrophages and the inhibition of inflammatory factor secretion by M1 macrophages in *vitro*

**Fig. 2:**
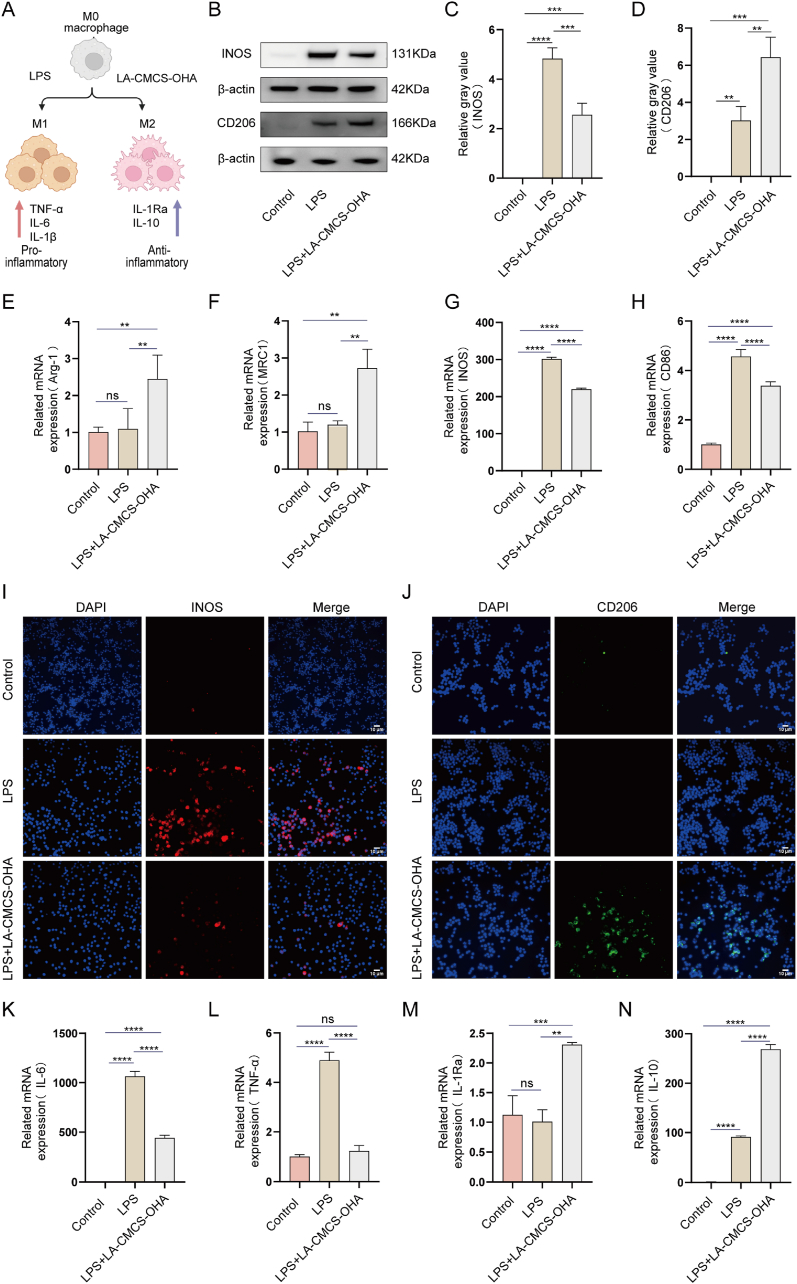
Effects of LA-CMCS-OHA Hydrogel on Macrophage Polarization and Secretion of inflammatory factors. (A) Schematic representation of macrophage polarization intervention with LPS and LA-CMCS-OHA. (B–D) Protein levels of iNOS and Arg-1 were assessed by Western blotting and semi-quantification. (E–H) mRNA levels of iNOS, CD86, Arg-1, and MRC1 were determined using RT-qPCR analysis. (I, J) Protein levels of iNOS and CD206 were evaluated by immunofluorescence. scale bar: 10 μm. (K–N) mRNA levels of IL-6, TNF-α, IL-10, and IL-1Ra were measured using RT-qPCR.

To evaluate the effect of LA-CMCS-OHA hydrogel on macrophages, macrophages were cultured in a medium containing LPS and LA-CMCS-OHA hydrogel to observe their polarization and cytokine secretion (Fig. 2A). Western blot analysis revealed that the hydrogel inhibited the expression of the M1 macrophage marker iNOS while enhancing the expression of the M2 macrophage marker CD206 (Fig. 2B–D). qRT-PCR results further demonstrated significant differences in the expression of surface markers for M1 macrophages (iNOS, CD86) and M2 macrophages (MRC1, Arg-1). The LA-CMCS-OHA hydrogel group showed substantial inhibition of iNOS and CD86 expression, accompanied by a marked increase in Arg-1 and MRC1 expression (Fig. 2E–H). Immunofluorescence analysis supported these findings, showing decreased iNOS expression and increased CD206 expression in the LA-CMCS-OHA hydrogel group (Fig. 2I and J; Supplementary Fig. 6A and B). These results indicate that the LA-CMCS-OHA hydrogel effectively promotes macrophage polarization towards the M2 phenotype.

qRT-PCR analysis revealed that the LA-CMCS-OHA hydrogel significantly reduced the secretion of pro-inflammatory cytokines, including TNF-α and IL-6, in M1 macrophages (Fig. 2K and L), while increasing the expression of anti-inflammatory cytokines IL-10 and IL-1Ra (Fig. 2M and N). Furthermore, ELISA results confirmed that the LA-CMCS-OHA hydrogel and its components, LA and CMCS-OHA, effectively decreased the secretion of pro-inflammatory cytokines such as IL-1β, IL-6, and TNF-α (Supplementary Fig. 6C–E). Collectively, these findings suggest that the LA-CMCS-OHA hydrogel promotes M2 macrophage polarization and mitigates the inflammatory response.

### 3.6 Mechanisms associated with macrophage polarization by LA-CMCS-OHA hydrogel

**Fig. 3:**
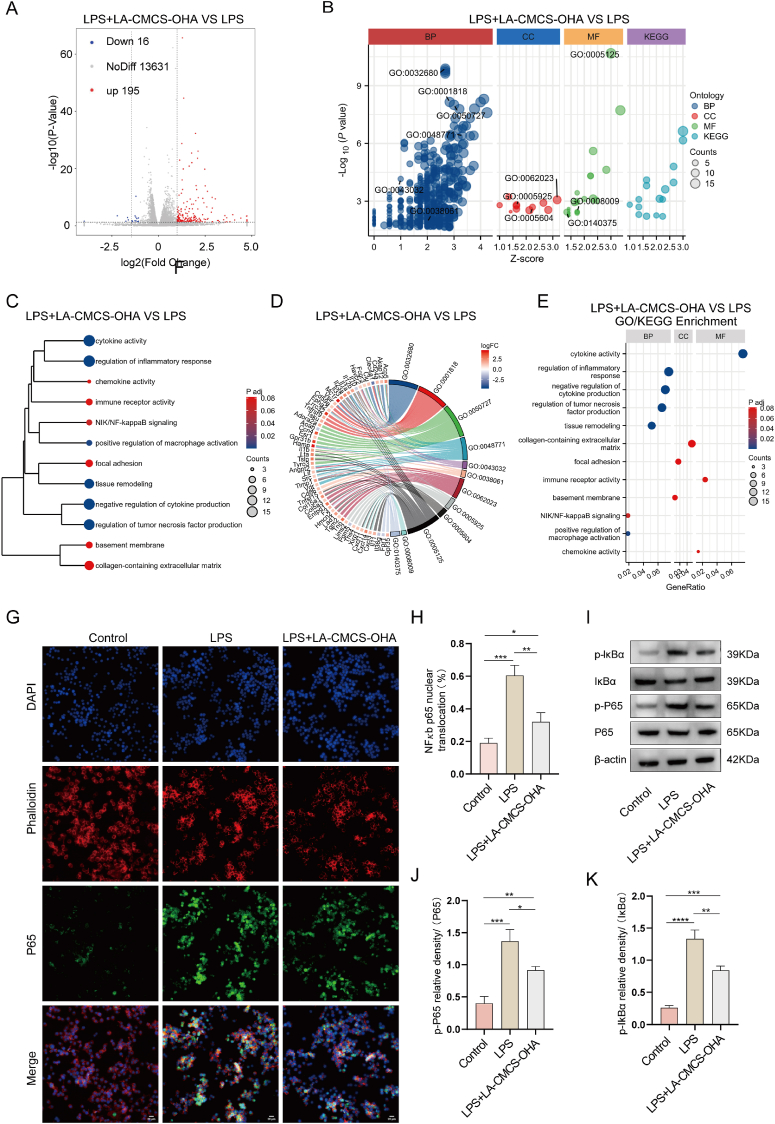
Transcriptome sequencing, immunofluorescence, and western blot analyses were conducted to investigate the mechanisms by which the LA-CMCS-OHA hydrogel regulates macrophage function. (A, B) Volcano plots and heatmaps illustrate differentially expressed genes between the LPS and LPS + LA-CMCS-OHA hydrogel groups. (C) Dendrogram analysis highlights differences in inflammatory responses, cytokine expression, and signaling pathways between these groups. (D, E) GO and KEGG enrichment analyses identify key differentially expressed genes and associated pathways. (F, G) Immunofluorescence staining and semi-quantitative analyses demonstrate NF-κB p65 translocation into the nucleus. scale bar: 20 μm (H–J) Western blot and semi-quantitative analyses reveal changes in the expression of NF-κB pathway-related proteins, including p-IκBα, IκBα, p-P65, and P65.

Previous studies have shown that the NF-κB signaling pathway is significantly upregulated in macrophages following lipopolysaccharide (LPS) treatment [46]. Inhibition of this pathway has been strongly associated with promoting macrophage polarization from the pro-inflammatory M1 phenotype to the anti-inflammatory M2 phenotype [47]. Preclinical studies in mice with conditional knockout of NF-κB activation demonstrated accelerated healing of rotator cuff tendinopathy, whereas activation of the IKKβ complex impeded the healing process [48]. To investigate whether the LA-CMCS-OHA hydrogel promotes macrophage polarization from M1 to M2 by inhibiting the NF-κB pathway, transcriptome sequencing was conducted on macrophages treated with LPS alone and LPS combined with LA-CMCS-OHA hydrogel. Principal Component Analysis (PCA) revealed distinct RNA expression profiles between the two groups (Supplementary Fig. 7A). Furthermore, a total of 211 differentially expressed genes were identified (Supplementary Fig. 7B). Upon LPS stimulation, macrophages polarized into the M1 phenotype, characterized by the upregulation of inflammation-related genes and pathways. Volcano and heatmaps clearly illustrated significant differences in gene expression between the LPS group and the LPS + LA-CMCS-OHA hydrogel group (Fig. 3A and B). Dendrogram analysis further highlighted a strong association with inflammatory responses (Fig. 3C).To explore the functional roles of these differentially expressed genes, GO enrichment and KEGG pathway analyses were conducted (Fig. 3D and E; Supplementary Fig. 7C and D). These analyses showed that the genes were primarily involved in cellular responses to LPS, immunomodulation, and inflammation-related pathways. The findings confirm that macrophages polarized to the M1 phenotype following LPS stimulation, with concurrent upregulation of inflammation-associated genes and pathways. Among these, the NF-κB pathway emerged as a pivotal regulator of inflammation and immunomodulation, playing a central role in apoptosis, stress responses, and other physiological processes, highlighting its potential as a key regulatory mechanism. Immunofluorescence staining revealed that the number of NF-κB p65 nuclear-localized macrophages after LPS stimulation was approximately three times higher than in the control group. However, the addition of LA-CMCS-OHA hydrogel reduced this number by about half (Fig. 3F and G).Western blotting analyses revealed a reduction in protein expression levels of p-P65/P65 and p-IκBα/IκBα in the LA-CMCS-OHA hydrogel group in comparison to the LPS group (Fig. 3H–J). These findings suggest that the LA-CMCS-OHA hydrogel mitigates the inflammatory response mediated by M1 macrophages by inhibiting the NF-κB pathway.

### 3.7 Promoting angiogenesis by LA-CMCS-OHA hydrogel *in vitro*

**Fig. 4:**
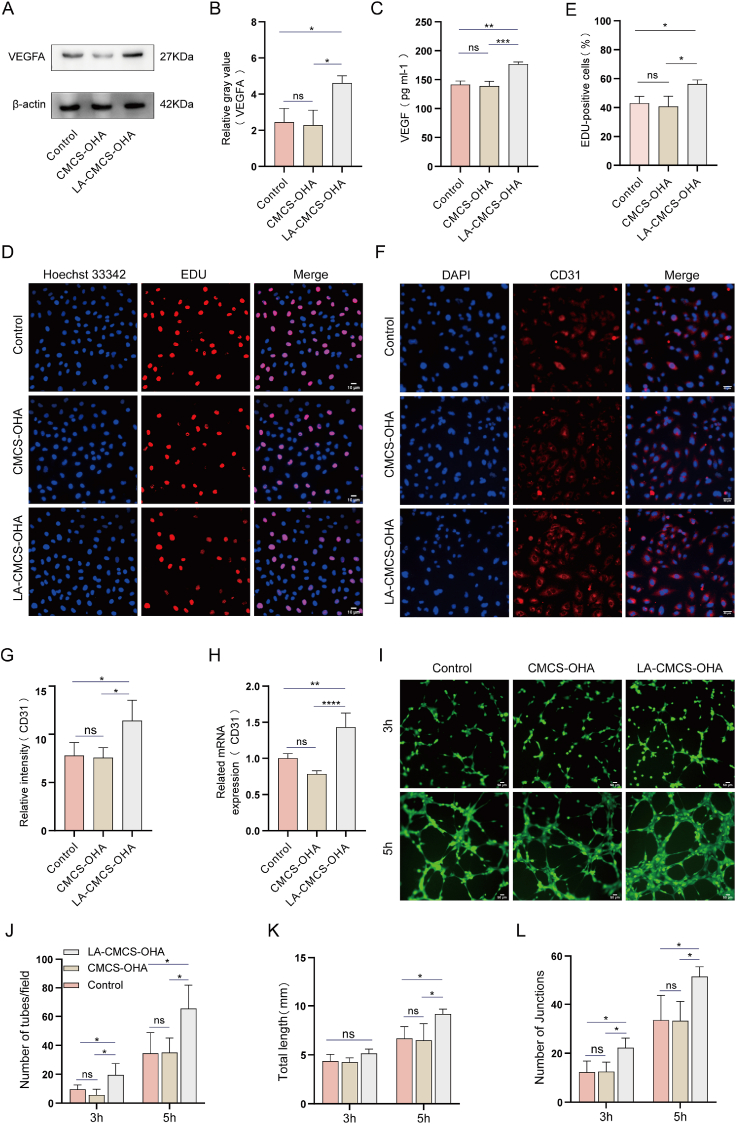
Evaluation of the vascularization potential of the LA-CMCS-OHA hydrogel in *vitro*. (A, B) VEGFA protein levels were analyzed via western blot and semi-quantification. (C) VEGF levels were quantitatively measured using ELISA. (D, E) HUVEC proliferation rates were assessed through EDU analysis of CMCS-OHA and LA-CMCS-OHA hydrogels. scale bar: 10 μm. (F, G) CD31 protein levels were analyzed using immunofluorescence and semi-quantification. scale bar: 10 μm. (H) CD31 mRNA levels were determined via RT-qPCR. (I–L) Tube formation assays were conducted at 3 and 5 h to evaluate differences in tube formation, total length, and the number of junctions among groups. scale bar: 10 μm.

VEGF and eNOS are critical angiogenic factors that stimulates the proliferation and migration of vascular endothelial cells, thereby promoting new blood vessel formation. Western blot analysis demonstrated a significant increase in VEGFA protein expression in HUVECs treated with the LA-CMCS-OHA hydrogel compared to both the control and CMCS-OHA hydrogel groups (Fig. 4A and B). Similarly, ELISA results indicated higher VEGF expression in the LA-CMCS-OHA hydrogel group compared to the control and CMCS-OHA hydrogel groups (Fig. 4C). Moreover, qRT-PCR analysis revealed elevated expression levels of both eNOS and VEGF in the LA-CMCS-OHA hydrogel group relative to other groups (Supplementary Fig. 8A and B).

The EDU results demonstrated a significantly increased cell proliferation rate in the LA-CMCS-OHA hydrogel group compared to the control and CMCS-OHA hydrogel groups (Fig. 4D and E). CD31 is a marker of vascular endothelialization [49]. Immunofluorescent staining confirmed elevated CD31 expression in HUVECs treated with the LA-CMCS-OHA hydrogel in comparison to the control and CMCS-OHA hydrogel groups (Fig. 4F and G), which is consistent with the results of the qRT-PCR analysis (Fig. 4H).

*In vitro* tube formation assays showed significantly enhanced angiogenesis in the LA-CMCS-OHA hydrogel group compared to the CMCS-OHA hydrogel and control groups at both the 3 h and 5 h time points (Fig. 4I–L). Quantitative analysis of tube formation parameters, including the number of nodes, total branch length, and number of tubes, confirmed significantly higher values in the LA-CMCS-OHA hydrogel group, indicating enhanced angiogenesis. Collectively, these findings suggest that the LA-CMCS-OHA hydrogel effectively promotes VEGF secretion, the proliferation and migration of vascular endothelial cells, and angiogenesis.

### 3.8 LA-CMCS-OHA hydrogel controls the early inflammatory response to RCTs

**Fig. 5:**
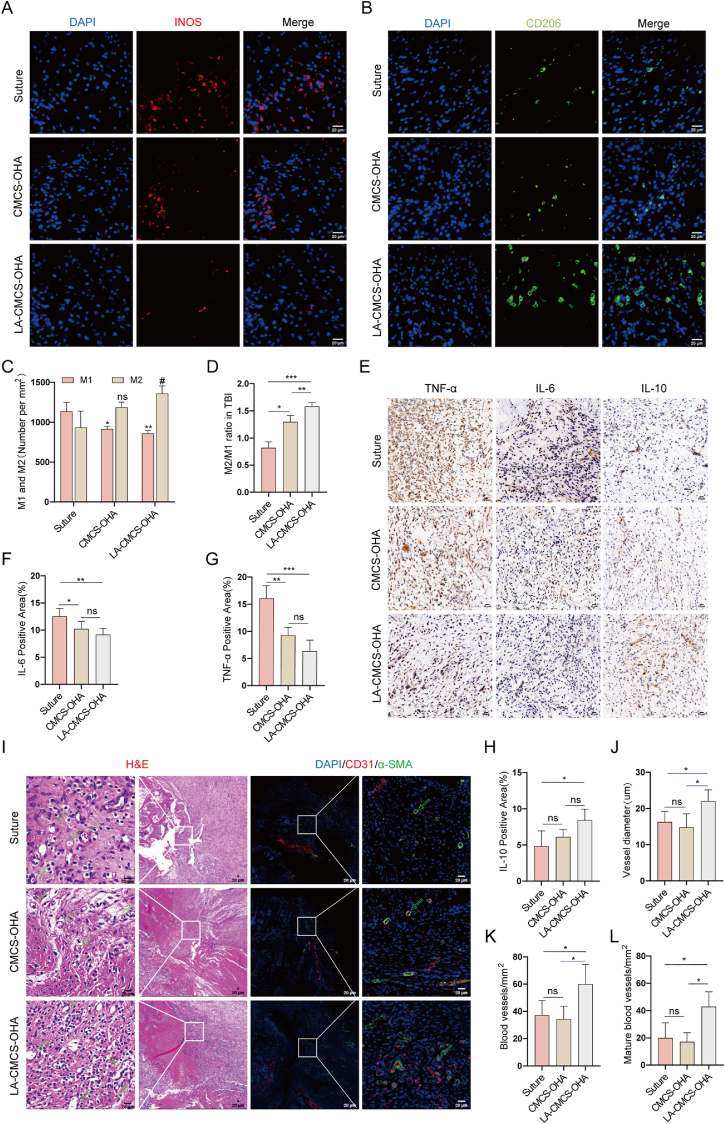
In *vivo* efficacy of the LA-CMCS-OHA hydrogel was evaluated in terms of its impact on macrophage polarization, the inflammatory response and angiogenesis.(A, B) Immunofluorescence analysis was performed to assess iNOS and CD206 expression levels at the rotator cuff TBI. scale bar: 20 μm. (C, D) Quantification of iNOS- and CD206-positive cells and the M2/M1 ratio. (E-G, I) Immunohistochemical and semi-quantitative analyses of IL-6, TNF-α, and IL-10. scale bar: 20 μm. (H) H&E staining and immunofluorescence analyses revealed the effects of the LA-CMCS-OHA hydrogel on blood vessel formation. Red arrows indicate neovascularization, while green arrows indicate mature vessels. scale bar: 20 μm. (J–L) Quantification of vessel diameter, total vessel count, and mature vessel count at the rotator cuff TBI. (For interpretation of the references to color in this figure legend, the reader is referred to the Web version of this article.)

In the early stages of RCTs, the immune system is activated, releasing pro-inflammatory factors that recruit and activate macrophages. These macrophages secrete large amounts of inflammatory mediators, promoting the formation and progression of an inflammatory microenvironment. This microenvironment disrupts cellular functions, induces metabolic abnormalities, and can lead to cell death. Prolonged exposure to this environment damages the cell's repair mechanisms, resulting in fibrosis, scarring, and ultimately weakening the tissue's regenerative capacity, potentially leading to further injury [50]. On the third postoperative day, immunofluorescence analysis revealed a notable presence of iNOS-positive cells at the TBI. In contrast, CD206-positive cells were barely detectable, indicating an impeded transition of macrophages from the M1 to M2 phenotype. Treatment with LA-CMCS-OHA hydrogel significantly enhanced the number of CD206-positive cells and the ratio of M2 to M1 macrophages at the site of TBI (Fig. 5A–D). Furthermore, immunohistochemical analysis demonstrated that post-treatment with LA-CMCS-OHA hydrogel markedly reduced the levels of pro-inflammatory cytokines IL-6 and TNF-α at the TBI (Fig. 5E–G), while significantly increasing the levels of the anti-inflammatory cytokine IL-10 (Fig. 5I). These outcomes highlight the substantial anti-inflammatory benefits of LA-CMCS-OHA hydrogel in the early postoperative period following RCT.

### 3.9 LA-CMCS-OHA hydrogel promotes angiogenesis in *vivo*

Newly formed blood vessels are essential for RCT repair. This is because the newly created blood vessels have the ability to provide adequate nutrients and minerals to the repair site [51]. Blood vessel formation was assessed in each group post-RCT surgery using H&E staining and immunofluorescence (Fig. 5H). 14 days after surgery. H&E staining showed a significantly larger blood vessel diameter in the LA-CMCS-OHA hydrogel group compared to the suture and CMCS-OHA hydrogel groups (Fig. 5J). CD31 and α-SMA double immunofluorescence staining further demonstrated that the number and maturity of blood vessels were significantly higher in the LA-CMCS-OHA hydrogel group than in the other groups (Fig. 5K and L). During rotator cuff repair, LA-CMCS-OHA hydrogel reduces inflammation in the torn area and creates a microenvironment favorable for the growth of endothelial cells, thus promoting neovascularization.

### 3.10 Histological analysis

**Fig. 6:**
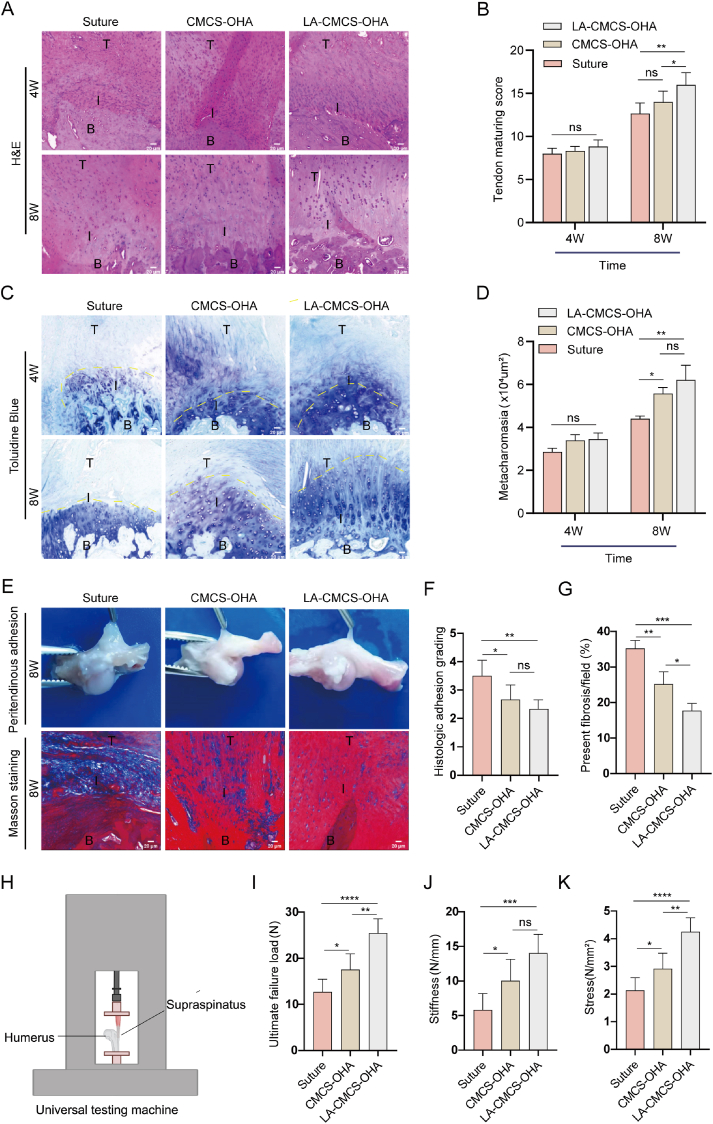
Histological analysis of the reconstructed TBI and biomechanical evaluation of the supraspinatus-humeral head complex. (A, B) Tendon maturity was assessed using H&E staining and quantitatively scored. scale bar: 20 μm. (C, D) Mineralization at the rotator cuff TBI was analyzed through toluidine blue staining and quantified. scale bar: 20 μm. (E) Fibrosis at the TBI was evaluated using macroscopic observation and Masson's trichrome staining. scale bar: 20 μm. (F) Peripheral adhesions were scored quantitatively. (G) The fibrosis area ratio was calculated. (H) Biomechanical testing of the supraspinatus humeral complex was performed using a universal testing machine. (I) The ultimate load to failure was measured. (J) Stress responses were assessed. (K) Stiffness was analyzed. T: tendon, I: interface, B: bone. (For interpretation of the references to color in this figure legend, the reader is referred to the Web version of this article.)

The effect of LA-CMCS-OHA hydrogel on the healing of RCTs was assessed using histopathological analysis of the TBI. H&E staining was performed on the interface tissue. At 4 weeks, none of the groups exhibited a healed TBI; instead, immature granulation tissue with sparse collagen was observed. Compared to the suture and CMCS-OHA hydrogel groups, the LA-CMCS-OHA hydrogel group displayed reduced inflammatory cell infiltration and a more organized tendon tissue structure. By the 8 week, the repair process in the LA-CMCS-OHA hydrogel group was nearly complete, displaying significantly more organized collagen fibers (Fig. 6A). Furthermore, enhanced tendon maturation was noted in this group compared to the suture and CMCS-OHA hydrogel groups (Fig. 6B), according to established tendon maturation scoring criteria (Supplementary Table 2–4).

Toluidine blue staining indicated an increase in fibrocartilage area over time. At 4 week, no significant differences in fibrocartilage area were observed among the groups. The tissue retained a heterogeneous morphology and remained immature. By week 8, the fibrocartilage area in the LA-CMCS-OHA hydrogel group had increased more than in the suture and CMCS-OHA groups (Fig. 6C and D). At this stage, the tissue structure had matured significantly, displaying normal cellular morphology and organization.

The RCT repair involves both exogenous and endogenous processes, with the exogenous process often resulting in extensive peritendinous adhesions, excessive collagen production, and irregular collagen alignment. Peritendinous adhesions were evaluated through direct observation (Supplementary Table 5). The administration of the LA-CMCS-OHA hydrogel at the site of the tear served to function as a barrier to adhesion to surrounding tissues. The surrounding tissue of the tendons in the LA-CMCS-OHA hydrogel group was observed to be smooth, easily manipulable with forceps, and free of visible adhesions within the peritendinous synovium. In contrast, the suture and CMCS-OHA hydrogel groups exhibited varying degrees of peritendinous adhesions (Fig. 6E and F).

Fibrosis in the rotator cuff was assessed using Masson's trichrome staining. The LA-CMCS-OHA hydrogel demonstrated significant anti-inflammatory properties, which reduced fibrosis associated with the inflammatory response. Compared to the suture and CMCS-OHA hydrogel groups, the LA-CMCS-OHA hydrogel group exhibited a lower fibrosis area ratio at the TBI and a more uniform arrangement of collagen bundles (Fig. 6E–G). Overall, these histopathological findings indicate that the LA-CMCS-OHA hydrogel effectively promotes the regeneration and repair of RCTs.

### 3.11 LACMCS-OHA hydrogel promotes RCT reconstruction with enhanced biomechanical strength

Biomechanical tests were conducted on different groups of the supraspinatus-humeral complex (Fig. 6H). 8 weeks post-treatment, no significant differences were observed in the cross-sectional area of the TBI (Supplementary Fig. 9). However, the LA-CMCS-OHA hydrogel group exhibited significantly superior biomechanical properties compared to both the suture and CMCS-OHA hydrogel groups. Specifically, the LA-CMCS-OHA hydrogel group demonstrated a substantially higher ultimate load to failure (25.47 ± 3.06 N) than the suture group (12.71 ± 2.74 N) and the CMCS-OHA hydrogel group (17.56 ± 3.40 N) (Fig. 6I). Similarly, the stiffness of the LA-CMCS-OHA hydrogel group was found to be significantly higher, with a mean value of (14.05 ± 2.69 N/mm), in comparison to the suture group (5.82 ± 2.37 N/mm) and the CMCS-OHA hydrogel group (10.05 ± 3.09 N/mm) (Fig. 6J). Furthermore, stress values were higher in the LA-CMCS-OHA hydrogel group (4.25 ± 0.51 N/mm^2^) compared to the suture group (2.13 ± 0.45 N/mm^2^) and the CMCS-OHA hydrogel group (2.91 ± 0.56 N/mm^2^) (Fig. 6K).These results suggest that the LA-CMCS-OHA hydrogel significantly enhances the biomechanical strength of the rotator cuff, providing nearly double the strength of control treatments.

The complexity of the pathological microenvironment is the main reason for the poor regenerative ability after RCT. Previous studies have primarily focused on tissue remodeling, whereas the pathological microenvironment has largely been overlooked. Current evidence indicates that suppression of the inflammatory response is essential for successful rotator cuff repairs for this study [25,52]. In contrast to growth factors and exosomes, LA, being a natural metabolite, exhibits low immunogenicity, low cost, and diverse biological activities. The controlled dynamic Schiff base reaction between CMCS and OHA offers a viable route for large-scale production. The synthesized hydrogel exhibits excellent biocompatibility and degradability. Our findings indicate that the hydrogel effectively facilitated the transformation of macrophages from the M1 to M2 phenotype through inhibition of the NF-κB signaling pathway. Furthermore, its excellent injectability enables minimally invasive drug delivery, thereby reducing surgical trauma and aligning well with clinical requirements.

Revascularization of the TBI is a critical factor contributing to rotator cuff healing [53]. Our experimental results revealed that the hydrogel promotes VEGF secretion, which enhances endothelial cell proliferation and migration, stimulates neovascularization, and provides crucial nutrient support, thereby accelerating tissue repair. However, excessive or sustained activation of the VEGF signaling pathway can lead to various pathological risks, including tumor progression and metastasis, pathologic vascular diseases, autoimmune disorders, and organ fibrosis and injury [54,55]. Consequently, precise regulation of VEGF signaling is crucial for maintaining vascular homeostasis and preventing associated pathologies. The LA-CMCS-OHA hydrogel developed in this study features a relatively short in vivo degradation period (approximately 7 days), thereby allowing the beneficial effects of VEGF to be harnessed while mitigating the pathological risks associated with prolonged signaling pathway activation. Several strategies are proposed for mitigating the potential risks associated with VEGF. A primary objective is the development of smart, responsive, tissue-engineered scaffolds that facilitate precise spatiotemporal control of VEGF delivery. Furthermore, combining VEGF therapies with targeted interventions, such as TGF-β inhibitors for fibrosis and vascular stabilizers to reduce leakage, offers another avenue for risk mitigation. Moreover, dynamic microenvironmental monitoring, employing advanced imaging techniques or validated biomarkers, can facilitate the assessment and management of these potential risks. Crucially, further investigation is required to thoroughly elucidate the mechanisms of VEGF action. This will facilitate the development of more precise and effective therapeutic strategies capable of addressing current clinical challenges.

Notwithstanding its limitations, this study offers considerable research value. A limitation is the inability to observe the dynamic distribution and degradation of the injected LA-CMCS-OHA hydrogel or monitor changes in LA concentration over time. Furthermore, this study employs a rat model of acute RCT. Clinically, RCTs are often associated with degenerative changes, and their complex pathology cannot be fully replicated in a rat model.The hydrogel presented in this study creates a favorable microenvironment for rotator cuff repair but has limited mechanical properties, precluding its use as a substitute for injured tendons or ligaments. Despite these shortcomings, the strategy proposed, involving the design of hydrogels with both anti-inflammatory properties and the capacity to promote neovascularization, offers considerable promise for clinical translation.

## 4 Conclusions

RCTs are challenging to treat due to their complex structure and intricate pathological processes. Tissue-engineered scaffolds represent a promising therapeutic approach for RCTs. In this study, we developed the LA-CMCS-OHA hydrogel, which modulated macrophage polarization by inhibiting the pro-inflammatory M1 phenotype and promoting the anti-inflammatory M2 phenotype. Moreover, the LA-CMCS-OHA hydrogel enhanced VEGF secretion, facilitating neovascularization. Furthermore, the LA-CMCS-OHA hydrogel is safe, injectable, and cost-effective, making it highly suitable for practical clinical application. In summary, the LA-CMCS-OHA hydrogel represents a novel therapeutic approach for RCTs with substantial potential for clinical translation.

## CRediT authorship contribution statement

**Bitao Wang:** Writing – original draft, Investigation. **Yiyang Hou:** Investigation, Data curation. **Xi Shang:** Data curation. **Yuxuan Zhou:** Visualization. **Yubiao Yang:** Software. **Zhenhan Li:** Visualization, Software. **Boyuan Ma:** Visualization. **Zhi Zeng:** Software. **Jinyu Chen:** Data curation. **Cheng Tang:** Data curation. **Jian Hao:** Writing – review & editing, Supervision. **Lianyong Wang:** Writing – review & editing, Supervision, Conceptualization. **Xianhu Zhou:** Project administration, Funding acquisition.

## Declaration of competing interest

The authors declare that they have no known competing financial interests or personal relationships that should be open.

## Data Availability

All relevant data supporting the findings of this study are either incorporated within the article and its supplementary information files or available upon request from the corresponding author.
