# Supplementary material for: Multifunctional hydrogel promotes rotator cuff healing through anti-inflammation and vascularization
Source: Mater Today Bio. 2025 Jun 23;33:102016. doi: 10.1016/j.mtbio.2025.102016 (PMC12268896; doi:10.1016/j.mtbio.2025.102016)
Supplement: Multimedia component 1 [file mmc1.docx]

**Multifunctional Hydrogel Promotes Rotator Cuff Healing Through Anti-Inflammation and Vascularization**

Bitao Wang ^a,#^, Yiyang Hou ^b,#^, Xi Shang ^c,#^, Yuxuan Zhou ^d^, Yubiao Yang ^e^, Zhenhan Li ^e^, Boyuan Ma^e^, Zhi Zeng^e^, Jinyu Chen^e^,  Cheng Tang ^e^, Jian Hao ^e ,^*,Lianyong Wang^b,^* , Xianhu Zhou^e,^ *

^a^ Ningbo University Health Science Center, No. 818, Fenghua Road, Jiangbei District, Ningbo, Zhejiang, China. 315211.

^b^ Key Laboratory of Bioactive Materials, Ministry of Education, College of Life Sciences, Nankai University, Tianjin China. 300071.

^c^ Taizhou Hospital of Zhejiang Province affiliated to Wenzhou Medical University, No.1 Tong Yang Road East, Luqiao District, Taizhou City, Zhejiang Province, China. 318050.

^d^ Portola High School, 1001 Cadence, Irvine, CA, 92618.

^e^ The Second Affiliated Hospital of Guangzhou Medical University, Guangzhou 510260.

# These authors contributed equally.

*Correspondence:

Xianhu Zhou,

No. 250, Changgang East Road, Haizhu District, Guangzhou City, Guangdong Province, China

Email: [zhouxianhu@gzhmu.edu.cn](mailto:zhouxianhu@gzhmu.edu.cn)

Lianyong Wang,

Key Laboratory of Bioactive Materials, Ministry of Education, College of Life Sciences, Nankai University, Tianjin China.

Email: wly@nankai.edu.cn

Jian Hao,

No. 250, Changgang East Road, Haizhu District, Guangzhou City, Guangdong Province, China

Email: [haojian@gzhmu.edu.cn](mailto:haojian@gzhmu.edu.cn)


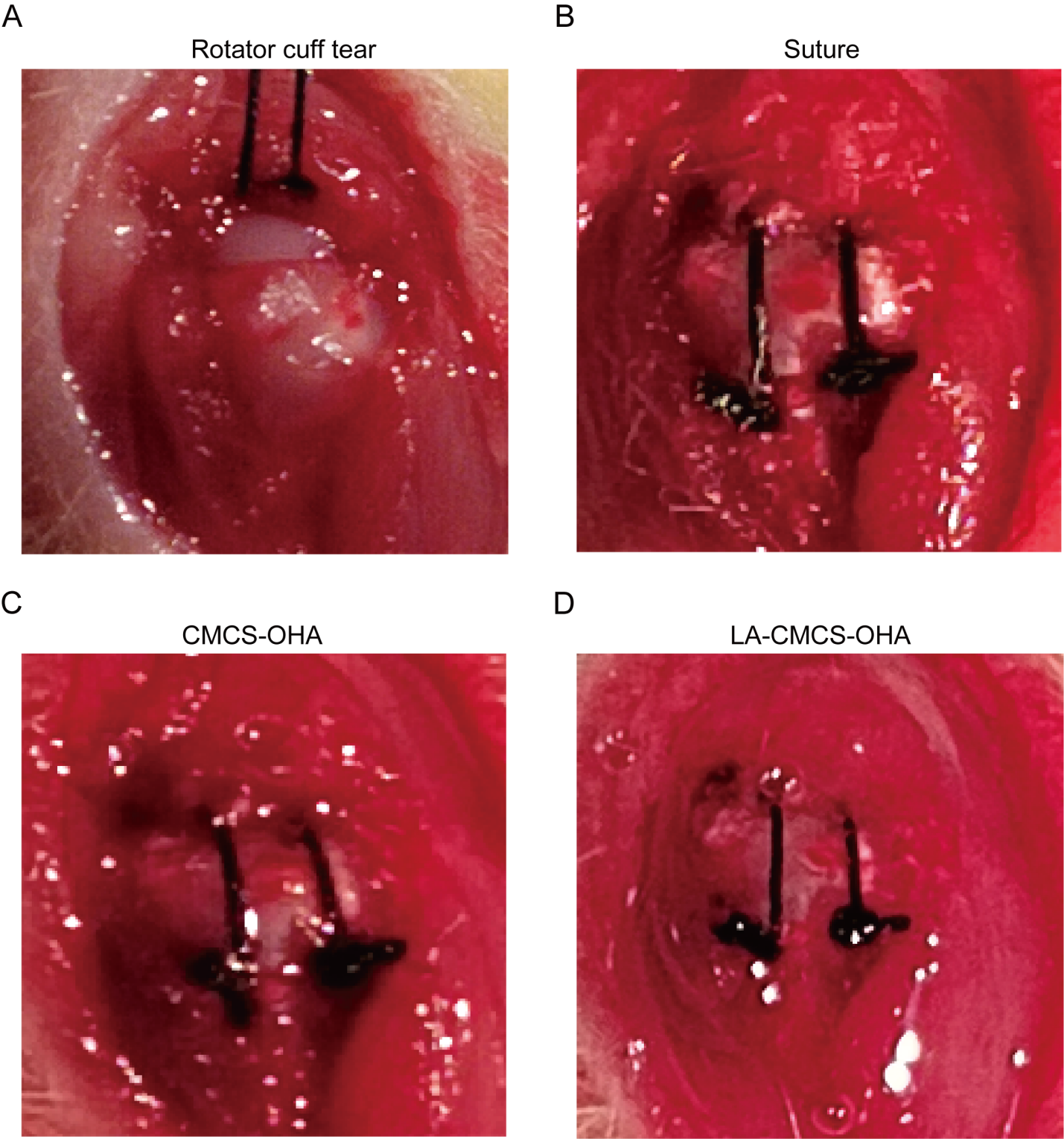


**Supplementary Figure 1.** Establishment of an acute RCT model repair in rats was achieved through a series of surgical procedures. (A) The supraspinatus tendon was surgically severed to mimic an acute injury. (B) Sutures were passed through the bone tunnel for fixation. (C, D) Either CMCS-OHA hydrogel or LA-CMCS-OHA hydrogel was administered to assess their respective therapeutic effects.


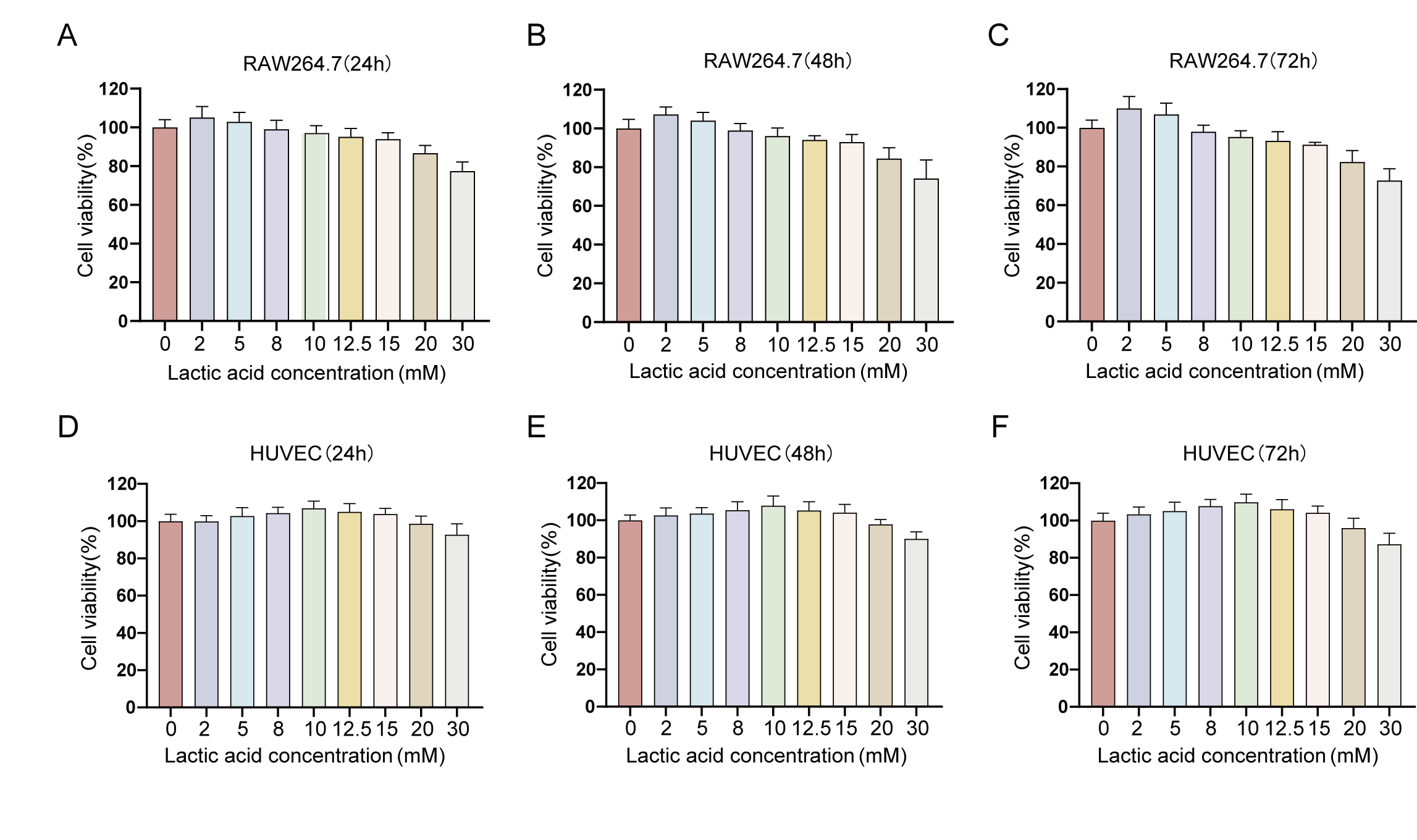


**Supplementary Figure 2.** Cytotoxicity assessment of LA. (A–C) CCK-8 proliferation assay of RAW264.7 cells cultured with varying LA concentrations on days 1, 2, and 3. (D–F) CCK-8 proliferation assay of HUVECs cultured with varying LA concentrations on days 1, 2, and 3.


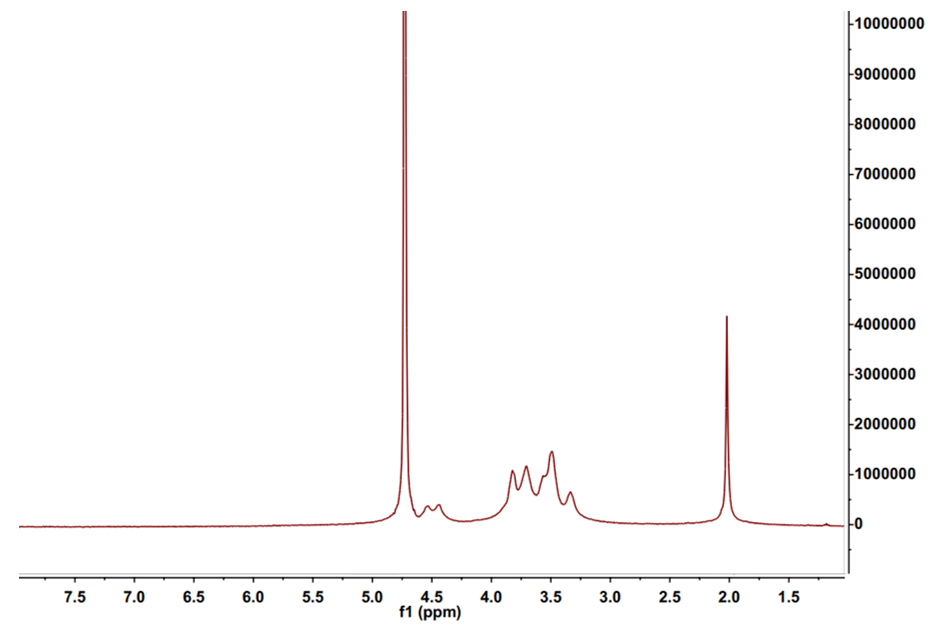


1. ^1^H NMR spectrum of HA


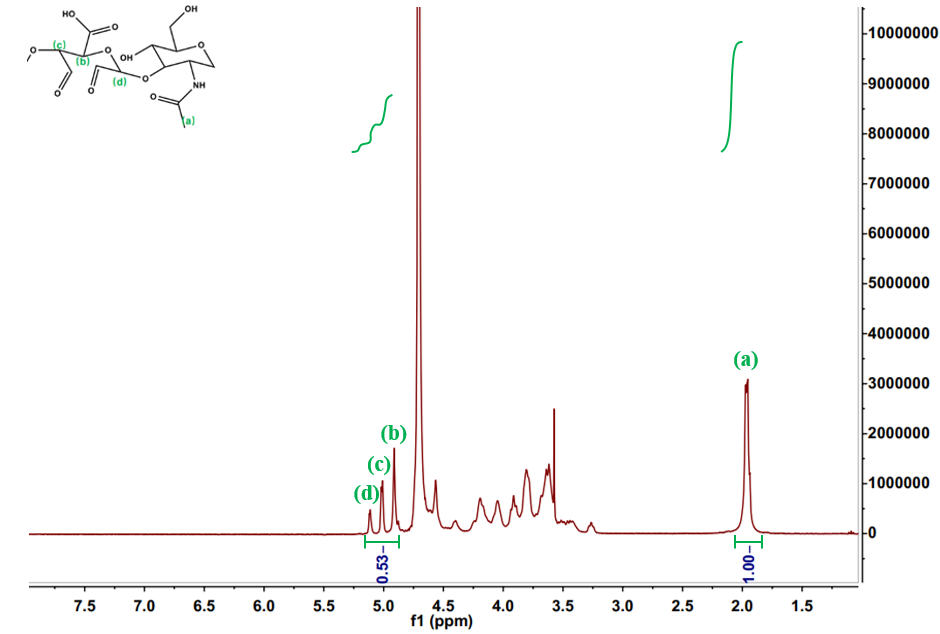


(B) ^1^H NMR spectrum of OHA

**Supplementary Figure 3.** (A)^1^H NMR spectrum of HA. (B) ^1^H NMR spectrum of OHA.


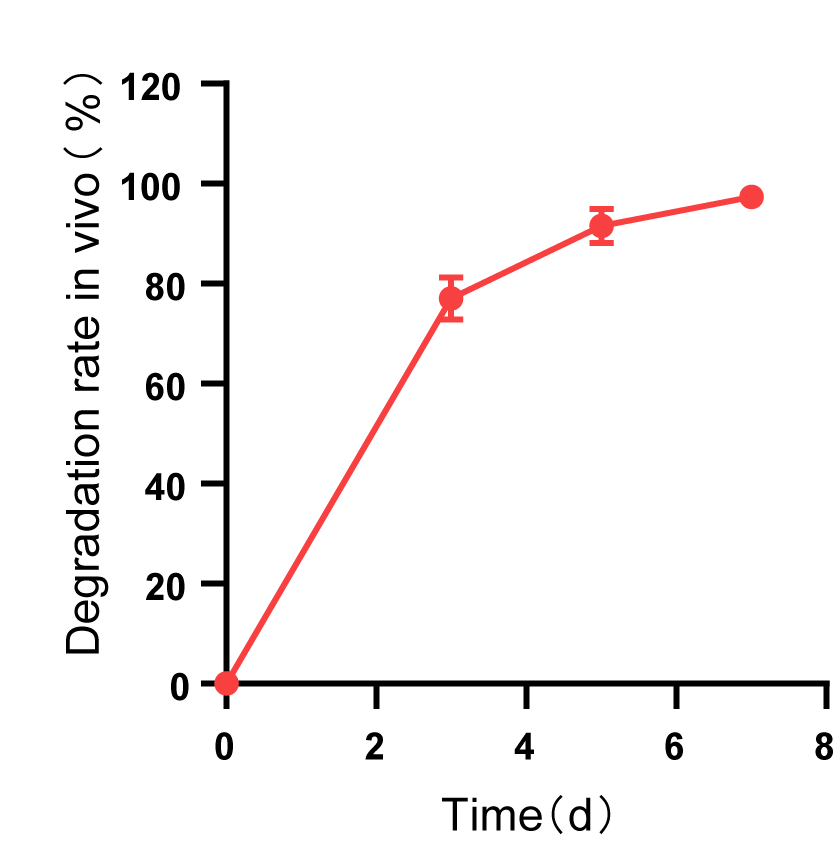


**Supplementary Figure 4.** In *vivo* degradation rate of LA-CMCS-OHA hydrogel.


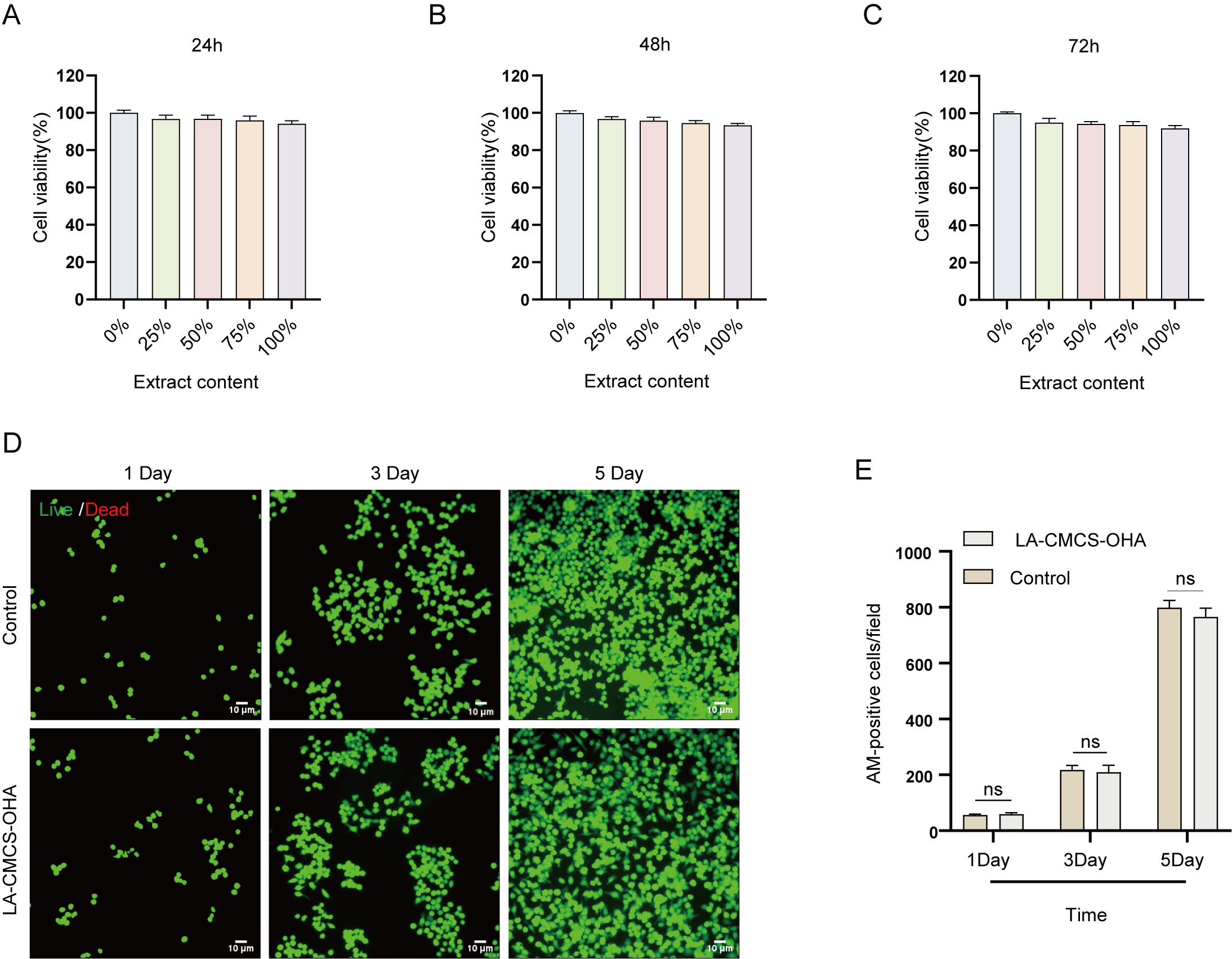


**Supplementary Figure 5.** Cytocompatibility of LA-CMCS-OHA hydrogel was assessed. (A-C) CCK8 assay results showed the proliferation of RAW264.7 cell after incubation with different concentrations of proportional LA-CMCS-OHA hydrogel for 1, 2, and 3 days. (D,E) Live/dead staining was performed on RAW264.7 cells after 1, 3, and 5 days of culture in LA-CMCS-OHA hydrogels.


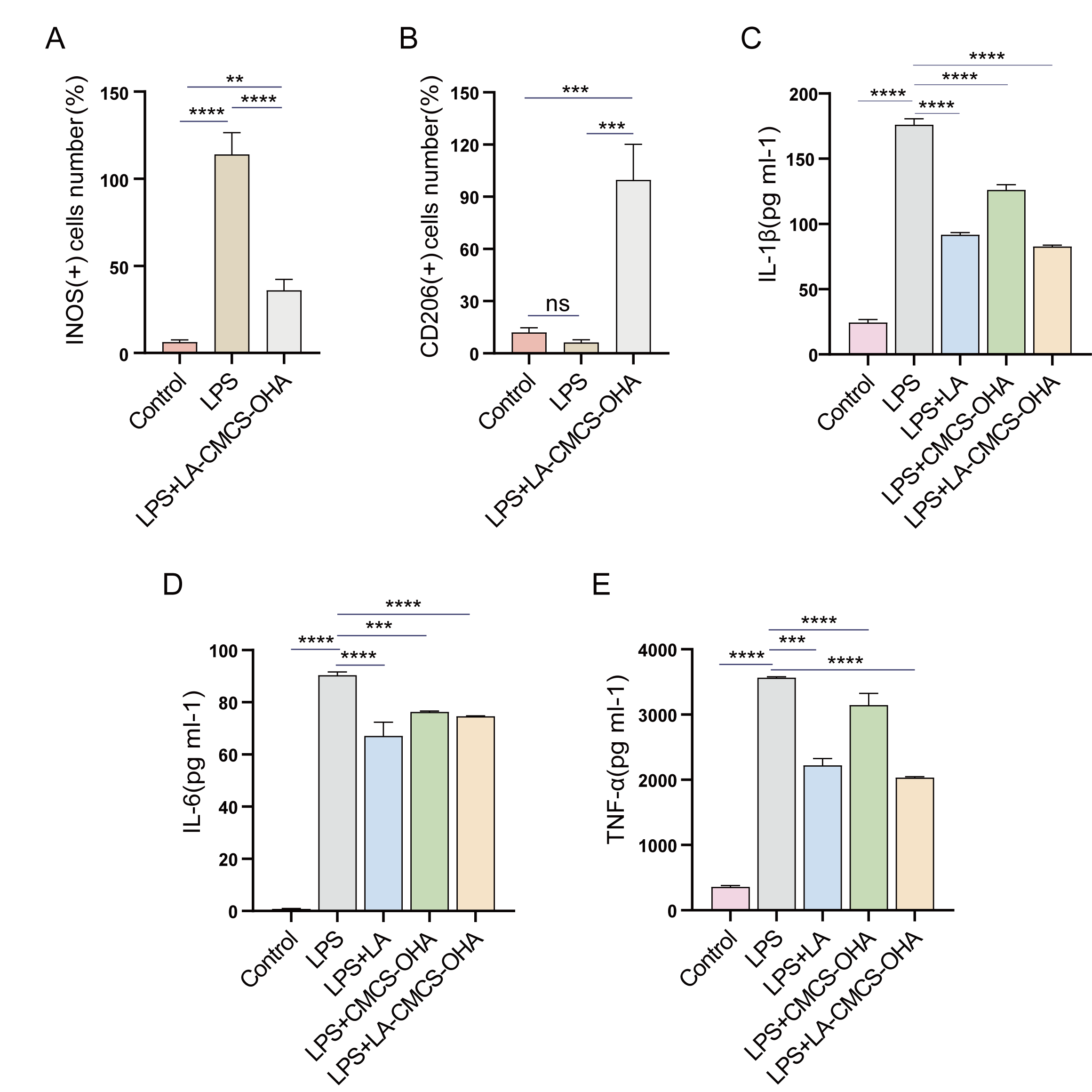


**Supplementary Figure 6.** LA-CMCS-OHA hydrogel regulates macrophage polarization and inhibition of secretion of inflammatory factors. (A,B)To evaluate the protein expression levels of iNOS and CD206, a semi-quantitative immunofluorescence assay was conducted. (C-E) The concentrations of IL-6, IL-1β, and TNF-α were quantified through ELISA.


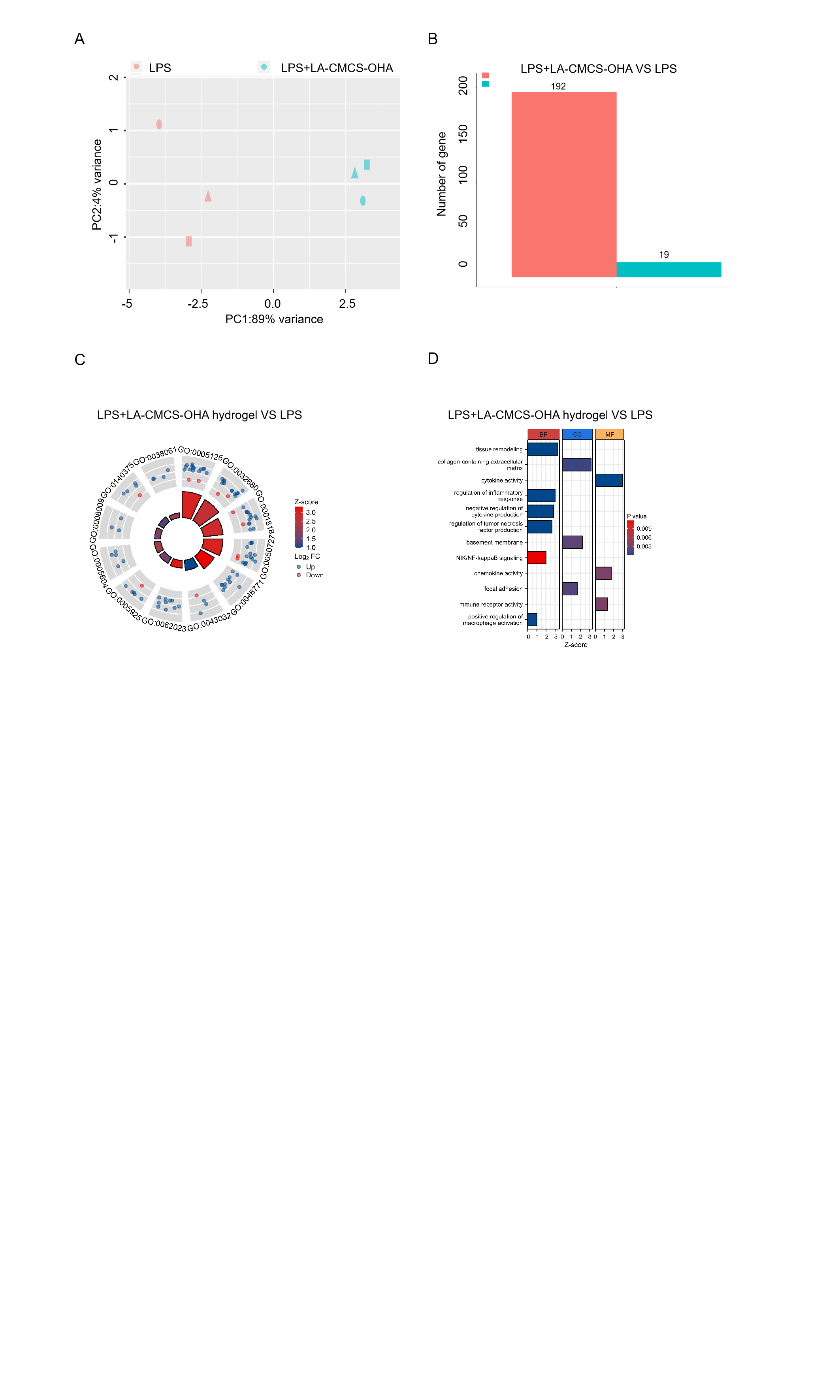


**Supplementary Figure 7.** Transcriptome sequencing was conducted to analyze macrophages treated with the LA-CMCS-OHA hydrogel. (A) Principal component analysis was performed on each group following Transcriptome sequencing to visualize variance among samples. (B) Histogram showing differential gene expression between LPS-treated and LPS+LA-CMCS-OHA hydrogel-treated groups. (C, D) GO and KEGG enrichment analyses were conducted to identify differentially expressed genes and associated pathways.


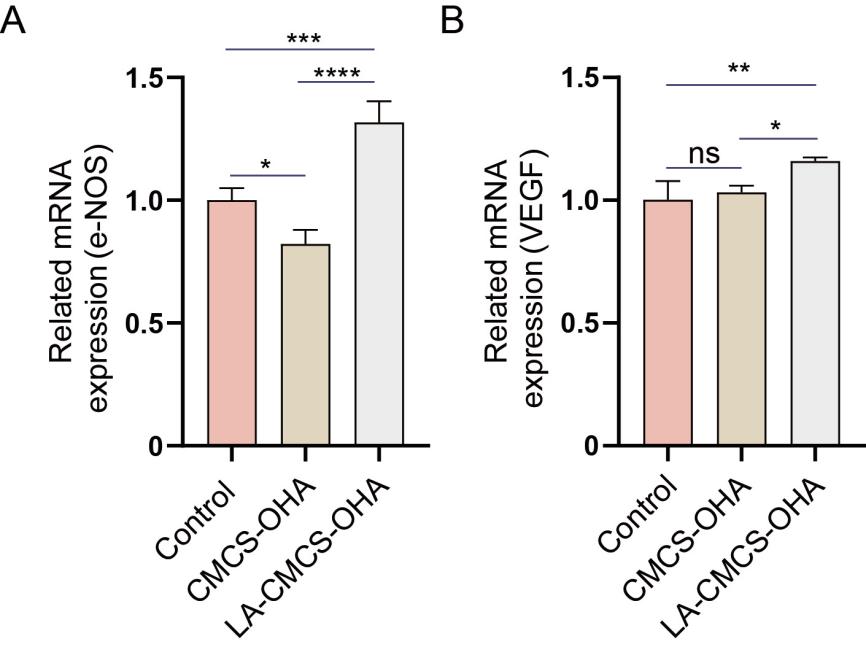


**Supplementary Figure 8.** Evaluation of vascularization of LA-CMCS-OHA hydrogel in vitro. (A,B)The mRNA levels of e-NOS and VEGF were analyzed using RT-qPCR.

**
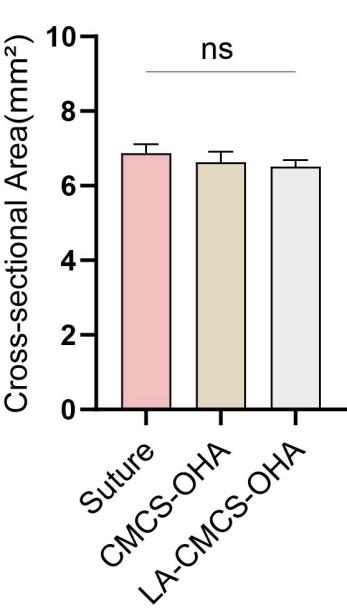
**

**Supplementary Figure 9.** The cross-sectional area of the tendon-bone interface was determined through the utilisation of physical measurements.

**Table 1.** Primers used for qRT-PCR.

| Abbreviation | Sequences | |
| --- | --- | --- |
|  | Forward | Reverse |
| m-β-actin | TGAGCTGCGTTTTACACCCT | GCCTTCACCGTTCCAGTTTT |
| m-MRC1 | GGCTGATTACGAGCAGTGGA | ACATGCCAGGGTCACCTTTC |
| m-ARG1 | ACATTGGCTTGCGAGACGTA | ATCACCTTGCCAATCCCCAG |
| m-INOS | GCCACCTCTACATTTGCGGA | CTGCTCCTCGCTCAAGTTCA |
| m-CD86 | TCAGTATCTCCAACAGCCTCTC | TCCAGAACACACACAACGGT |
| m-IL-10 | CCCTTTGCTATGGTGTCCT | GTGGCCAGTTTGTTATTTAT |
| m-IL-1Ra | CGTGTGCAGTCTCCAGAATATG | AAGAACACATTCCGAAAGTCAATAGG |
| m-TNF-α | GTTCCCAAATGGCCTCCC | GTGCTCCTCACCCACACCG |
| m-IL-6 | CTGCAAGAGACTTCCATCCAG | AGTGGTATAGACAGGTCTGTTGG |
| h-CD31 | CACCTCCAGCCAACTTCACCAT | CACTGTCCGACTTTGAGGCTATCT |
| h-eNOS | AGACCCACTGGTGTCCTCTT | CACACAGAACCTGAGGGTGC |
| h-VEGF | TGCGGATCAAACCTCACCA | CAGGGATTTTTCTTGTCTTGCT |

**Table 2.** Modified histological scoring system of the regenerated rotator cuff

| Modified tendon-maturing score | 1 | 2 | 3 | 4 |
| --- | --- | --- | --- | --- |
| Cellularity | Marked | Moderate | Mild | Minimal |
| Tendon-like cells, % | <25 | 25–50 | 50–75 | >75 |
| Cells in parallel, % | <25 | 25–50 | 50–75 | >75 |
| Fiber orientation, % | <25 | 25–50 | 50–75 | >75 |
| Interface histological findings | C+, R− | C+, R+ | C+, R+, F− | C+, R+, F+, tidemark+ |
| C, continuity; F, fibrocartilage; R, regularity, + positive; −, negative | | | | |

**Table 3.** Histological scores of the rotator cuff were evaluated in the suture, CMCS-OHA,

and LA-CMCS-OHA groups 4 weeks post-repair.

| Variable (mean score) | Suture | CMCS-OHA | LA-CMCS-OHA |
| --- | --- | --- | --- |
| Cellularity (1–4) | 1.83±0.41 | 1.83±0.41 | 1.5±0.5 |
| Tendon-like cells (1–4) | 1.33±0.52 | 1.83±0.41 | 1.67±0.47 |
| Cells in parallel (1–4) | 1.67±0.52 | 1.67±0.52 | 1.83±0.37 |
| Fiber orientation (1–4) | 1.83±0.41 | 1.5±0.55 | 2.0±0 |
| Interface histological findings (1–4) | 1.33±0.42 | 1.5±0.55 | 1.83±0.37 |
| Total score (5–20) | 8.0±0.63 | 8.33±0.52 | 8.83±0.69 |

**Table 4.** Histological scores of the rotator cuff were evaluated in the suture, CMCS-OHA,

and LA-CMCS-OHA groups 8 weeks post-repair.

| Variable (mean score) | Suture | CMCS-OHA | LA-CMCS-OHA |
| --- | --- | --- | --- |
| Cellularity (1–4) | 2.33±0.52 | 2.67±0.52 | 2.67±0.52 |
| Tendon-like cells (1–4) | 2.17±0.41 | 2.5±0.55 | 3.17±0.41 |
| Cells in parallel (1–4) | 2.67±0.52 | 2.67±0.52 | 3.33±0.51 |
| Fiber orientation (1–4) | 2.67±0.52 | 2.83±0.41 | 2.83±0.41 |
| Interface histological findings (1–4) | 2.83±0.41 | 3.33±0.52 | 3.0±0.70 |
| Total score (5–20) | 12.67±1.21 | 14±1.26 | 16±1.41 |

**Table 5 .** Histologic grading of peritendinous adhesions

| The histological evaluation standards for peritendinous adhesion. The histological scoring adhesion tissue was evaluated into Grades 1-5. |
| --- |
| Grade 1, no adhesions; |
| Grade 2, adhesion tissue holding less than 25% in the peritendinous area of the repaired site; |
| Grade 3, adhesion tissue holding less than 25%~50% in the peritendinous area of the repaired site; |
| Grade 4, adhesion tissue holding 50%~75% in the peritendinous area of the repaired site; |
| Grade 5, adhesion tissue holding more than 75% in the peritendinous area of the repaired site. |
